# Thrombospondin-4 deletion does not exacerbate muscular dystrophy in β-sarcoglycan-deficient and laminin α2 chain-deficient mice

**DOI:** 10.1038/s41598-024-65473-8

**Published:** 2024-06-26

**Authors:** Paula Zarén, Kinga I. Gawlik

**Affiliations:** Muscle Biology Unit, Department of Experimental Medical Science, Lund University, BMC C12, 221 84 Lund, Sweden

**Keywords:** Diseases, Cell biology, Cell adhesion, Cell death, Mechanisms of disease, Membrane trafficking

## Abstract

Muscular dystrophy is a group of genetic disorders that lead to muscle wasting and loss of muscle function. Identifying genetic modifiers that alleviate symptoms or enhance the severity of a primary disease helps to understand mechanisms behind disease pathology and facilitates discovery of molecular targets for therapy. Several muscular dystrophies are caused by genetic defects in the components of the dystrophin-glycoprotein adhesion complex (DGC). Thrombospondin-4 overexpression has been shown to mitigate dystrophic disease in mouse models for Duchenne muscular dystrophy (dystrophin deficiency) and limb-girdle muscular dystrophy type 2F (LGMD2F, δ-sarcoglycan deficiency), while deletion of the thrombospondin-4 gene exacerbated the diseases. Hence, thrombospondin-4 has been considered a candidate molecule for therapy of muscular dystrophies involving the DGC. We have investigated whether thrombospondin-4 could act as a genetic modifier for other DGC-associated diseases: limb-girdle muscular dystrophy type 2E (LGMD2E, β-sarcoglycan deficiency) and laminin α2 chain-deficient muscular dystrophy (LAMA2-RD). Deletion of the thrombospondin-4 gene in mouse models for LGMD2E and LAMA2-RD, respectively, did not result in worsening of the dystrophic phenotype. Loss of thrombospondin-4 did not enhance sarcolemma damage and did not impair trafficking of transmembrane receptors integrin α7β1 and dystroglycan in double knockout muscles. Our results suggest that thrombospondin-4 might not be a relevant therapeutic target for all muscular dystrophies involving the DGC. This data also demonstrates that molecular pathology between very similar diseases like LGMD2E and 2F can differ significantly.

## Introduction

Muscular dystrophy is a heterogeneous group of degenerative inherited disorders that lead to muscle function failure. Progressive muscle wasting, often starting from a young age, results in compromised mobility, breathing difficulties and feeding complications^1^. To this end, mutations in more than 60 genes have been implicated to cause muscular dystrophy, giving rise to broad clinical spectra of the disease^2^. Although U.S. Food and Drug Administration has approved the first gene therapy for some patients with Duchenne muscular dystrophy^3^, currently there are no specific cures available to these severe and complex disorders. Development of therapy is hindered by the fact that most muscular dystrophy subtypes are rare diseases^2^.

The connection between the cytoskeleton, transmembrane receptor complexes and the extracellular matrix is important for muscle homeostasis and function. This is underscored by the fact that some muscular dystrophy variants are caused by mutations in genes that are a part of attachment networks^4^. The dystrophin-glycoprotein complex (DGC) constitutes a major adhesion complex in skeletal muscle. It is composed of intracellular components dystrophin, syntrophins and α-dystrobrevin that link cytoskeleton with transmembrane proteins dystroglycan (α- and β), sarcoglycans (α-, β-, δ-, γ- and ζ) and sarcospan^4,5^. α-dystroglycan binds to several extracellular matrix ligands, including the laminin α2 subunit of the laminin-211 heterotrimer^6,7^.

Mutations in the genes encoding the components of the DGC and the DGC ligand laminin α2 give rise to following muscle diseases: Duchenne/Becker muscular dystrophy (dystrophin mutation); limb-girdle muscular dystrophy (LGMD) type 2D, 2E, 2F and 2C (also called sarcoglycanopathies, caused by mutations in α-, β-, δ- and γ-sarcoglycan, respectively) and LAMA2-related muscular dystrophy (LAMA2-RD) (mutations in the laminin α2 chain gene)^2,4,5^.

Sarcoglycanopathies represent the most severe forms of LGMDs. They are rare autosomal recessive disorders and constitute about 10–25% of LGMDs worldwide^8^. The frequency of the individual subtypes varies significantly between different populations, with estimated prevalence between 0.27 and 0.56/100.000^8–10^ (prevalence of LGMD2E in Italian population is estimated at 0.86/1.000.000^11^). Weakness of proximal muscles occurs in early childhood and eventually leads to loss of ambulation and variable range of respiratory insufficiency^8,9,11–13^. Cardiac involvement is common in patients with LGMD2E^11^.

Severe form of LAMA2-RD, a congenital variant, is one of the most frequent among congenital muscular dystrophies (CMD), but prevalence variability in the different populations also needs to be considered. It accounts for about 10–30% of CMD cases in European countries (estimated prevalence 0.7–1.1/100.000)^14–16^ and 36% cases in China^17^. The majority of LAMA2-RD patients display severe phenotype^15^. Clinical manifestations are represented by neonatal onset of muscle weakness, delayed motor development, joint contractures, respiratory difficulties, and progressive weakness that prevents ambulation^15,18–21^. The considerable frequency of subclinical cardiac involvement has been reported^15^. Premature death in early teens occurs in half of patients^22^.

Animal models for sarcoglycanopathies and LAMA2-RD exist, and they adequately mirror the disease course in humans. They have served as an important tool for understanding the disease mechanisms and test treatments^9,23–26^. β-sarcoglycan-deficient mouse (*Sgcb* mouse), a model for LGMD2E, is characterized by loss of muscle force, cardiomyopathy, and elevated creatine kinase levels in plasma. The histopathological features of *Sgcb* muscle include extensive focal necrosis, inflammation, degeneration/regeneration cycles, variability of muscle fiber size, dystrophic calcifications, fibrosis and fatty infiltration^27–29^.

*Dy*^3*K*^*/dy*^*3K*^ mouse model for LAMA2-RD displays complete deficiency of laminin α2 chain. Loss of laminin α2 chain results in the most severe phenotype among mouse models lacking proteins of cell adhesion complexes^30^. The mouse suffers from growth retardation, progressive muscle waste and dramatic weight loss, and survives up to 5 weeks of age^25,31^. Atrophy of muscle fibers, degeneration/regeneration cycles, apoptosis, inflammation, and fibrosis are among the hallmarks of the disease in *dy*^3*K*^*/dy*^*3K*^ muscle^25,31–33^.

Because the DGC provides critical structural support to muscle cell membrane, loss of any of its components leads to sarcolemma instability and rupture^4,34–36^. Furthermore, loss of each DGC component results in destabilization of the entire complex^5,28,36,37^, which further aggravates sarcolemma fragility. Subsequent influx of calcium ions into muscle cell disrupts muscle homeostasis and ultimately leads to necrosis^38,39^. Cell death triggers inflammation and muscle repair, but muscle regeneration is either inefficient or eventually exhausted. As a result, muscle tissue is replaced by fibrotic lesions and fatty deposits.

Laminin α2 chain absence does not lead to drastic destabilization of the sarcolemma^34^. Molecular mechanisms involved in degeneration of laminin α2 chain-deficient muscle have not been entirely characterized. Loss of interaction between laminin α2 subunit and its receptors dystroglycan and integrin α7β1 leads to impaired signalling, which most likely results in increased protein degradation and decreased survival of muscle cells^4,33,40^. Additionally, impaired mitochondrial function and bioenergetic status could also contribute to muscle degeneration^41^.

Several studies in mouse models for the DGC-related muscular dystrophies have highlighted the role of genetic modifiers in modulation of disease phenotype^42–45^. Thrombospondin-4 (Thbs4) belongs to a family of glycoproteins involved in tissue remodelling upon injury^46,47^. In skeletal muscle it regulates the composition of the extracellular matrix, metabolism, and physiology; particularly in muscles with high content of oxidative fibers^48^. Overexpression of Thbs4 in dystrophin-deficient *mdx* mouse and δ-sarcoglycan-deficient *Sgcd* mouse mitigated dystrophic symptoms^43^. Consequently, deletion of the Thbs4 gene in these mouse models, exacerbated dystrophic phenotype^43^ and enhanced cardiomyopathy^49^. The mechanism behind phenotype modulation by Thbs4 was attributed to enhancement of cellular trafficking of the adhesion complexes to stabilize sarcolemma and boost muscle integrity. Hence, it has been proposed that Thbs4 is an attractive therapeutic target for muscular dystrophies involving mutations in the DGC^43^.

Here we aimed at verifying whether Thbs4 could influence the phenotype of other muscular dystrophies associated with the DGC. For that purpose, we deleted the Thbs4 gene in β-sarcoglycan knockout mouse (*Sgcb* KO, the mouse model for LGMD2E), and in a mouse model for LAMA2-RD (laminin α2 chain-deficient *dy*^*3K*^*/dy*^*3K*^ mouse). We found no exacerbation of the dystrophic phenotype in respective double knockout models (*Sgcb/Thbs4* and *dy*^*3K*^/*Thbs4*). This indicates a non-protective role of Thbs4 in these two muscle disorders. Thbs4 was upregulated in *dy*^*3K*^*/dy*^*3K*^ and *Sgcb* KO muscle, but only in the extracellular compartment and not intracellularly as described for other muscular dystrophy mouse models. Hence, the molecule could contribute to fibrotic phenotype rather than directly supporting sarcolemma stability through increased intracellular trafficking mechanisms.

## Results

### Deletion of Thbs4 does not exacerbate muscle function in mice with β-sarcoglycan deficiency

**Figure 1:**
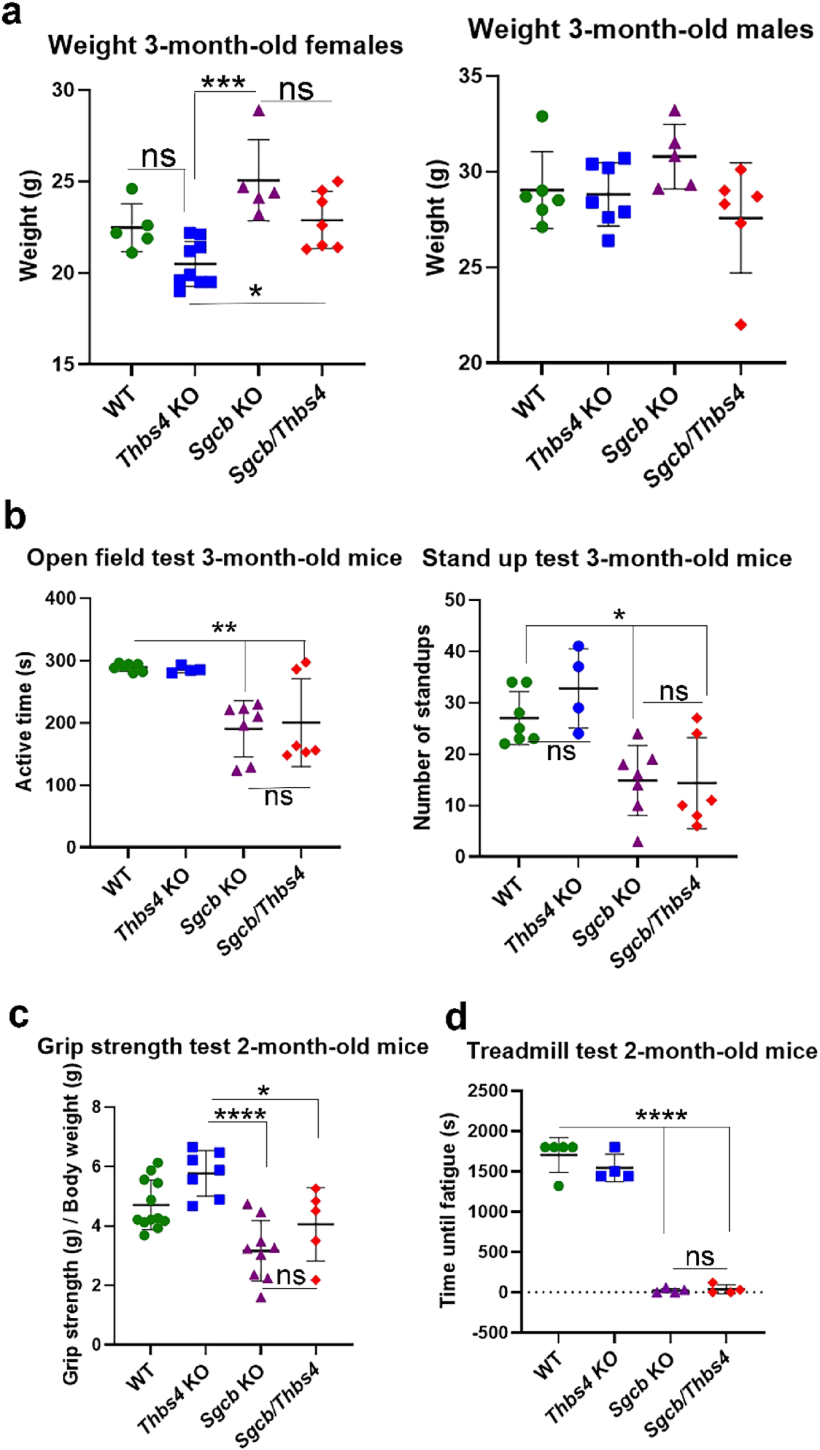
Deletion of thrombospondin-4 does not exacerbate outward signs of muscular dystrophy and does not worsen muscle function in mice with β-sarcoglycan deficiency. (**a**) Body weight analysis of 3-month-old wild-type (WT), *Thbs4* KO, *Sgcb* KO, *Sgcb/Thbs4* in males and females. There is a trend for increased weight in *Sgcb* KO females (p = 0.0697 WT vs *Sgcb* KO; p = 0.0002 *Thbs* KO vs *Sgcb* KO). Also, *Sgcb/Thbs4* females are heavier than *Thbs4* KO mice (p = 0.0279). Such differences were not noted in males. No significant difference in body weight were observed between *Sgcb* KO and *Sgcb/Thbs4* neither in females (p = 0.1105) nor in males (p = 0.0883). (**b**) Open field test and stand-up test carried out on 3-month-old WT, *Thbs4* KO, *Sgcb* KO and *Sgcb/Thbs4* mice. *Sgcb* KO and *Sgcb/Thbs4* mice performed significantly poorer than WT mice in the open field test (p = 0.0020 and p = 0.0075, respectively) and in the stand-up test (p = 0.0214 and p = 0.0211, respectively), but no significant difference can be observed between *Sgcb* KO and *Sgcb/Thbs4* mice in neither the open field test (p = 0.9947) nor the stand-up test (p = 0.9991). (**c**) Grip strength test performed in 2-month-old WT, *Thbs4* KO, *Sgcb* KO and *Sgcb/Thbs4* mice. Two-month-old dystrophic mice are weaker than control mice (p = 0.0043, *Sgcb* KO vs WT; p = 0.0.134, *Sgcb* KO vs *Thbs4* KO; p = 0.0208, *Sgcb/Thbs4* vs *Thbs4* KO). There are no significant differences between *Sgcb* KO and *Sgcb/Thbs4* mice. (**d**) Treadmill test of 2-month-old WT, *Thbs4* KO, *Sgcb* KO and *Sgcb/Thbs4* mice. *Sgcb* KO and *Sgcb/Thbs4* mice performed significantly poorer than healthy mice (p < 0.0001), but no significant difference was observed between *Sgcb* KO and *Sgcb/Thbs4* mice (p = 0.9989). All statistical analyses were performed using one-way ANOVA followed by Tukey’s multiple comparison test.

To assess how Thbs4 deletion would affect the general phenotype of β-sarcoglycan-deficient mice, we analyzed body weight and physical performance of single knockout mice (*Sgcb* KO) and double knockout mice (*Sgcb/Thbs4*) as well as control groups (wild-type (WT) and *Thbs4* KO). *Sgcb* KO mice show outwards signs of muscular dystrophy manifested with muscle hypertrophy and increased body weight at approximately two months of age^27^. We only observed mildly increased weight in *Sgcb* KO females at three months of age (Fig. 1a). Importantly, significant differences between *Sgcb* KO and *Sgcb/Thbs4* (in both males and females) were not observed at this age (Fig. 1a).

To assess how Thbs4 deletion affects muscle strength and physical capacity in mice with β-sarcoglycan deficiency, *Sgcb* KO, *Sgcb/Thbs4*, WT and *Thbs4* KO mice were subjected to open field activity test, stand-up test, grip strength test and treadmill training. The open field test and the stand-up test at 3 months of age showed differences between the control groups (WT, *Thbs4* KO) and the dystrophic groups (*Sgcb* KO and *Sgcb/Thbs4*): dystrophic mice performed significantly poorer than healthy mice, but there was no difference between *Sgcb* KO and double knockout animals (Fig. 1b). The grip strength test at 2 months of age revealed a similar trend: dystrophic mice were weaker than controls, but muscle strength of *Sgcb/Thbs4* mice was not decreased compared to *Sgcb* KO mice (Fig. 1c).

It has previously been shown that δ-sarcoglycan-deficient mice (*Sgcd*) lacking Thbs4 display reduced physical capacity compared to single-knockout *Sgcd* mice when subjected to forced treadmill running^43^. To assess if this also applies in β-sarcoglycan-deficiency, we subjected 2-month-old mice to treadmill running. Dystrophic mice performed significantly poorer than their age-matched healthy controls, as neither *Sgcb* KO nor *Sgcb/Thbs4* mice were able to run for more than a few minutes (Fig. 1d). There was no difference in running time between *Sgcb* KO and *Sgcb/Thbs4* (Fig. 1d). These results suggest that outward phenotype and muscle function in *Sgcb* KO and *Sgcb/Thbs4* are equally affected.

### Sarcolemmal stability is not impaired with Thbs4 deletion in β-sarcoglycan-deficient mice

**Figure 2:**
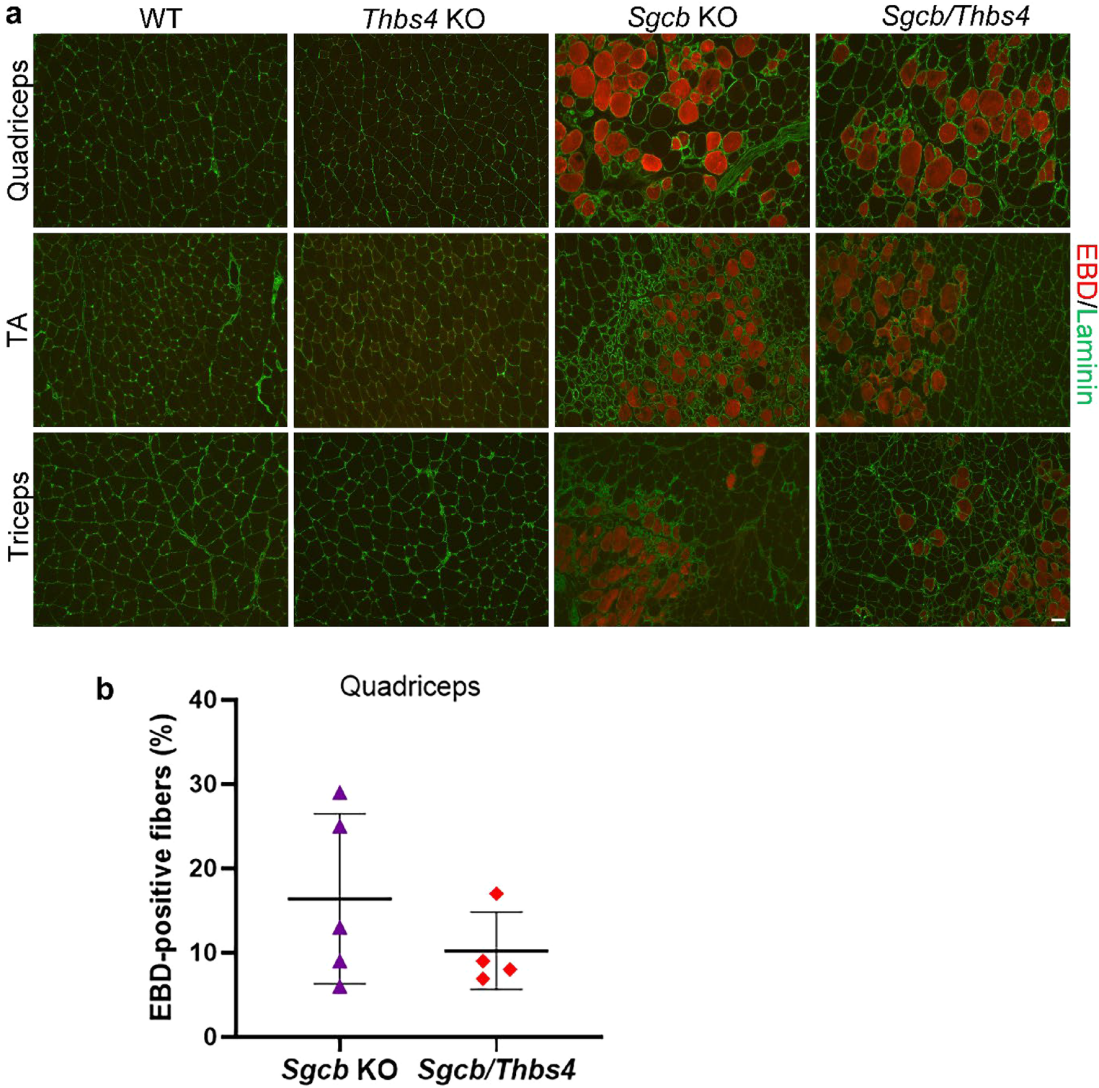
Sarcolemmal stability is not further compromised by thrombospondin-4 deletion in β-sarcoglycan-deficient mice. (**a**) Evans blue dye (EBD) staining of quadriceps, tibialis anterior (TA) and triceps muscle of 2-month-old WT, *Thbs4* KO, *Sgcb* KO, and *Sgcb/Thbs4* mice. EBD uptake (in red) in myofibers of *Sgcb/Thbs4* muscles is not visibly increased compared to myofibers of *Sgcb* KO muscles. Laminin staining (in green) visualizes muscle fibers. Bar, 50 μm. (**b**) Quantification of EBD-positive myofibers in quadriceps muscles of 2-month-old *Sgcb* KO and *Sgcb/Thbs4*. No significant difference was found between *Sgcb* KO and *Sgcb/Thbs4* muscle (p = 0.2986, t-test).

It has previously been shown that *Sgcd* mice lacking Thbs4 exhibited greater sarcolemmal damage in myofibers after physical exercise, indicating that loss of Thbs4 in these muscles decreases sarcolemma stability^43^. Because neither *Sgcb* KO nor *Sgcb/Thbs4* were able to run on treadmill, we could not compare the excess of sarcolemma rupture after training. However, even untrained *Sgcb* KO mice display substantial muscle cell membrane tearing^28,29^. Thbs4 deletion did not result in increased sarcolemma damage in untrained double knockout mice compared with *Sgcb* mice: we did not observe a greater uptake of Evans blue dye (EBD) in quadriceps, tibialis anterior, and triceps or muscles of double knockout mice (Fig. 2a). Quantification of EBD-positive cells in quadriceps muscle confirmed these observations (Fig. 2b), indicating that Thbs4 absence does not influence sarcolemmal stability in β-sarcoglycan-deficient muscle.

### Deletion of Thbs4 does not exacerbate pathohistological features of muscular dystrophy in β-sarcoglycan-deficient muscles

**Figure 3:**
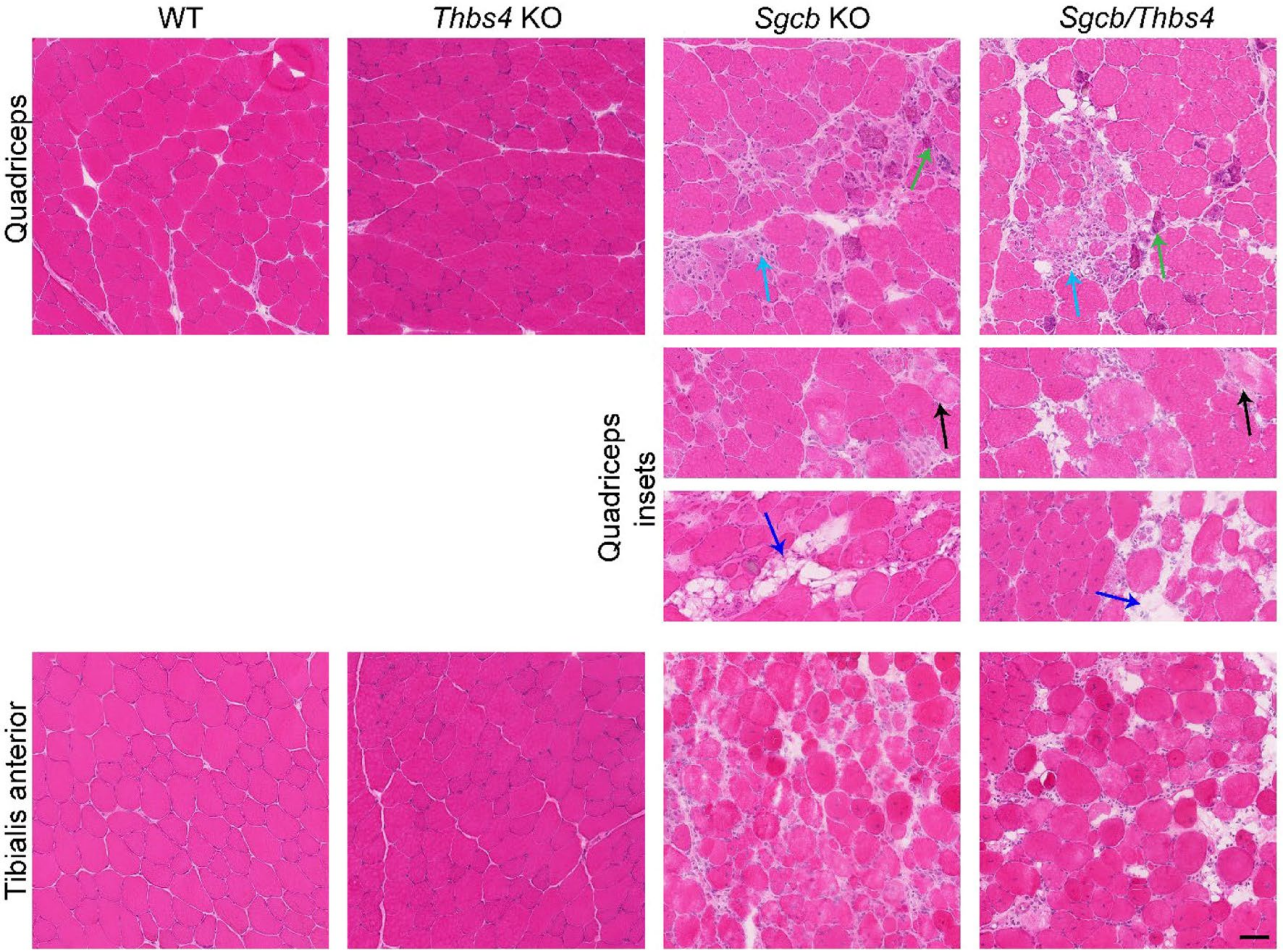
Deletion of Thbs4 does not exacerbate pathohistological features of muscular dystrophy in β-sarcoglycan-deficient muscles. Hematoxylin and eosin staining of quadriceps and tibialis anterior isolated from 3-month-old WT, *Thbs4* KO, *Sgcb* KO and *Sgcb/Thbs4* mice. *Thbs4* KO muscle does not exhibit histopathological signs of muscular dystrophy. Dystrophic mice (*Sgcb* KO and *Sgcb/Thbs4*) show histopathological signs of muscular dystrophy: necrotic cells (black arrows), inflammation (light blue arrows), regeneration (fibers with centrally located nuclei), calcification (green arrows), fatty tissue deposits (dark blue arrows). No difference regarding the degree of dystrophic changes was seen between *Sgcb* KO and *Sgcb/Thbs4* muscle. At least five mice from each group were analyzed. Bar: 50 μm.

Skeletal muscles of *Sgcb* KO mice already at young age show dystrophic morphological changes: central nucleation, fatty infiltration, necrosis and calcification^27,28^. Also, fibrosis is a feature of *Sgcb-null* muscle, which is especially pronounced in aged muscles^28,29^. To evaluate whether Thbs4 deletion worsens dystrophic features in *Sgcb* KO muscle, we performed hematoxylin and eosin staining of different muscles (quadriceps, tibialis anterior, triceps and diaphragm) isolated from 3-month-old WT, *Thbs4* KO, *Sgcb* KO, and *Sgcb/Thbs4* mice. *Thbs4* KO muscle did not exhibit any signs of muscular dystrophy in the examined muscles (Fig. 3 and Supplemental Fig. S1). A distinct increase of central nucleation, number of necrotic cells, fat cell infiltration, and calcification was seen in *Sgcb* KO and in double knockout muscle compared to healthy controls (Fig. 3 and Supplemental Fig. S1). Both *Sgcb* KO and *Sgcb/Thbs4* muscles were severely affected by morphological dystrophic changes, but no differences regarding the dystrophic features were observed between these two groups (Fig. 3 and Supplemental Fig. S1).

**Figure 4:**
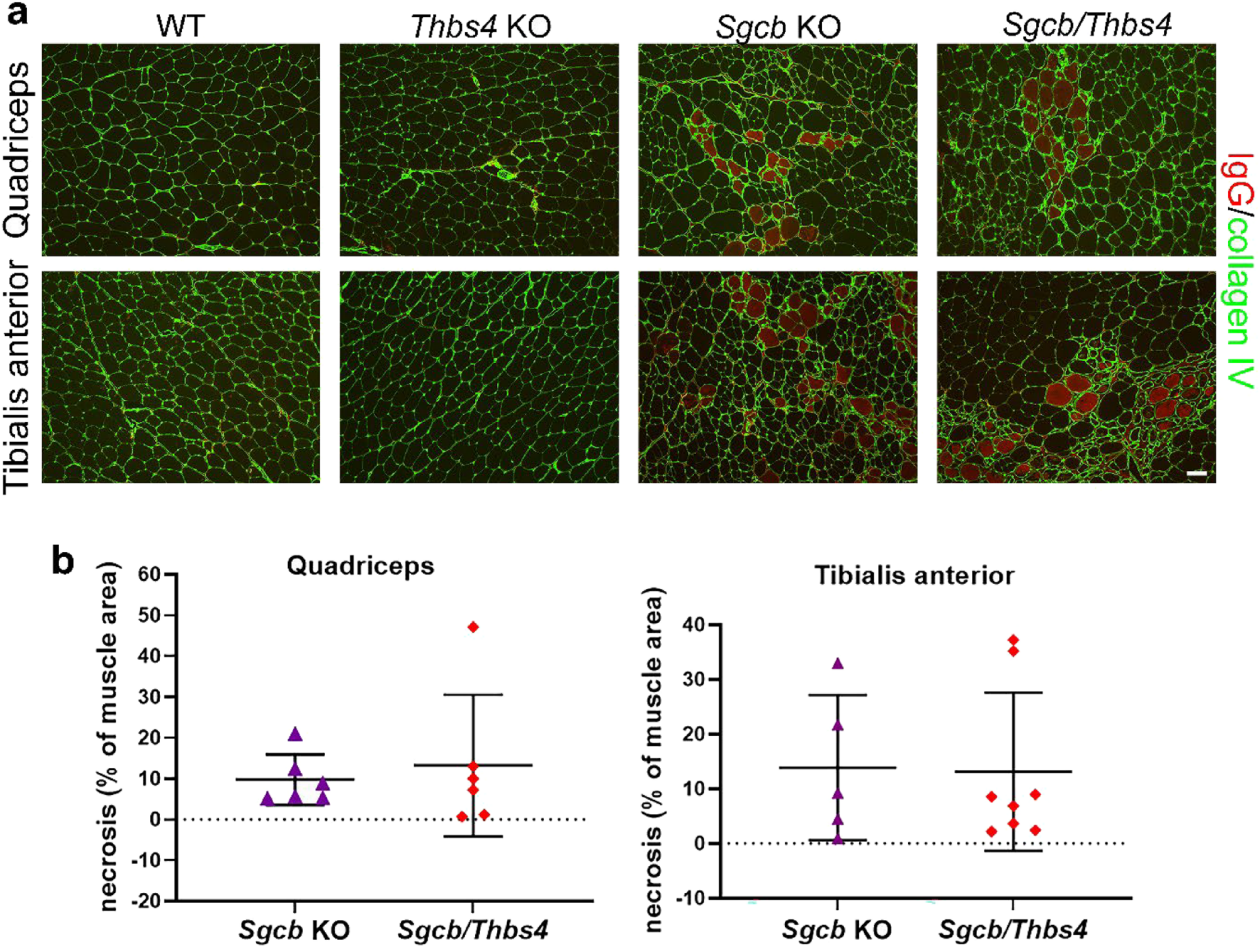
Deletion of Thbs4 does not increase necrosis and inflammation in *Sgcb* muscle. (**a**) Immunostaining with mouse IgG antibody (red) shows necrotic lesions/inflammatory sites in quadriceps and tibialis anterior muscles from 3-month-old dystrophic animals. No such lesions were detected in *Thbs4* KO muscle. Staining for collagen IV (in green) delineates muscle fibers. Bar: 50 μm. (**b**) Quantification of necrotic areas in quadriceps and tibialis anterior of *Sgcb* and *Sgcb/Thbs4* mice shows no significant difference between groups for both muscles (p = 0.6563 and p = 0.9285, student’s t-test).

This was confirmed by analysis of necrosis and inflammation. *Sgcb* and double knockout muscles (quadriceps and tibialis anterior) displayed large areas occupied with necrotic and inflammatory cells, as demonstrated by staining against mouse IgG^50–52^ (Fig. 4a). Quantification of necrosis and inflammation showed large variation between dystrophic animals from the same group, but in general no significant difference between single knockout and double knockout animals was demonstrated (Fig. 4b). It has previously been shown that deletion of Thbs4 drastically increased fibrotic build-up in δ-sarcoglycan-deficient skeletal muscles^43^. Collagen III immunostaining demonstrated onset of fibrosis in 3-month-old *Sgcb* and *Sgcb/Thbs4* muscles and visualized equal presentation of fibrotic material in both groups (Supplementary Fig. S2).

Heart muscle in *Sgcb* mouse shows pronounced necrosis and fibrosis from 5 months of age^28^. In 3-month-old *Sgcb* KO mice no cardiomyopathic changes could be detected (Supplementary Fig. S1), which stays in agreement with previous studies, where *Sgcb* hearts were reported to develop only mild cardiac abnormalities at young age^28^. No pathological changes were found in 3-month-old *Sgcb/Thbs4* heart muscle (Supplementary Fig. S1). This indicates that absence of Thbs4 does not cause acceleration of cardiomyopathy in *Sgcb* KO mice.

### Muscular dystrophy does not worsen with age in β-sarcoglycan double knockouts compared to β-sarcoglycan single knockouts

**Figure 5:**
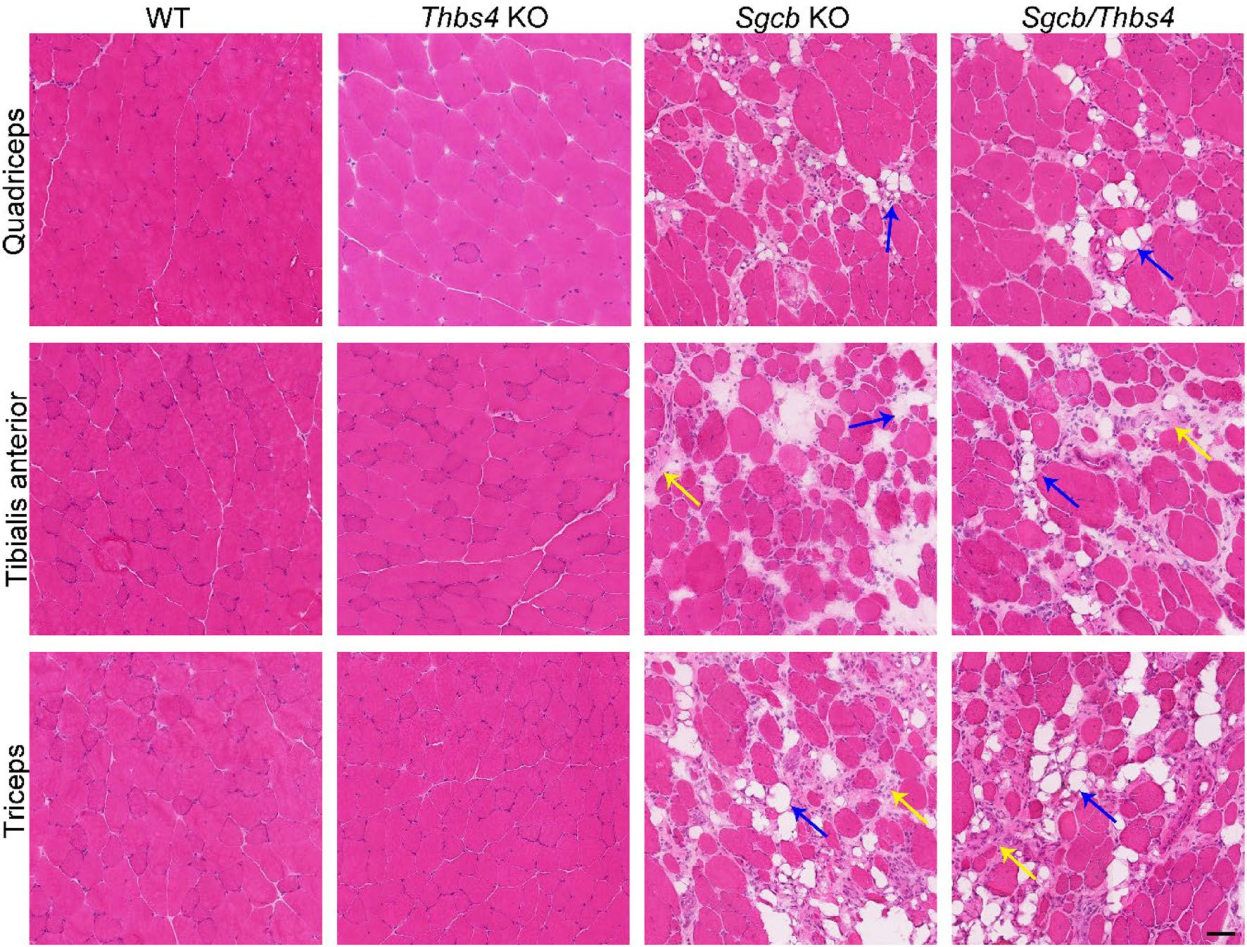
Deletion of Thbs4 does not enhance muscular dystrophy in aged *Sgcb* muscle. Hematoxylin and eosin staining of quadriceps femoris, tibialis anterior and triceps brachii isolated from 15-month-old WT, *Thbs4* KO, *Sgcb* KO and *Sgcb/Thbs4* mice. *Thbs4* KO muscle does not exhibit histopathological signs of muscular dystrophy. *Sgcb* KO and *Sgcb/Thbs4* muscles display equally pronounced fatty infiltrates (dark blue arrows) and fibrosis (yellow arrows). At least three mice from each group were analyzed. Bar, 50 μm.

It has previously been shown that at one year of age, *Thbs4* KO mice exhibit increased histopathological signs of muscular dystrophy, as well as reduced physical capacity^43^. Hence, it has been interpreted that loss of Thbs4 predisposes to muscular dystrophy. We examined older mice to explore whether β-sarcoglycan-deficient mice lacking Thbs4 would exhibit worsening of muscular dystrophy with age compared to *Sgcb* KO mice. Hematoxylin and eosin staining of quadriceps, tibialis anterior, and triceps muscles from 15-month-old WT, *Thbs4* KO, *Sgcb* KO and *Sgcb/Thbs4* mice was performed. Surprisingly, we did not find clear morphological signs of muscular dystrophy in *Thbs4* KO muscles (Fig. 5). Moreover, we did not observe a difference between *Sgcb* KO and *Sgcb/Thbs4* mice regarding histopathological signs of muscular dystrophy (Fig. 5). Both β-sarcoglycan-deficient muscle and double knockout muscle exhibited massive infiltration of fatty tissue (Fig. 5). This was also confirmed by Oil Red O staining that visualizes fat (Supplementary Fig. S3). We did not detect a difference in the range of fatty infiltrates between *Sgcb* KO and *Sgcb/Thbs4* muscle.

**Figure 6:**
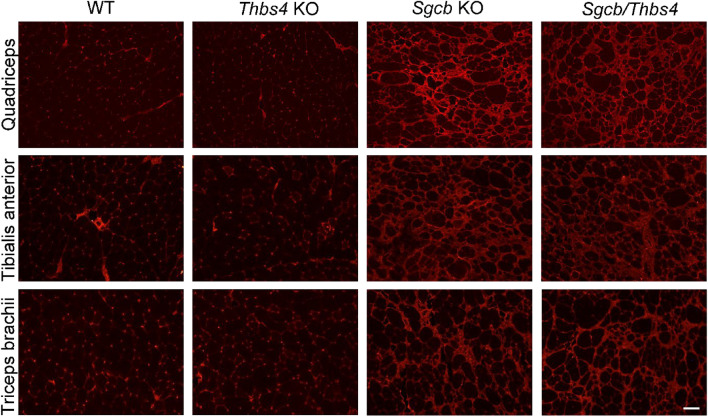
Fibrosis is not enhanced in *Sgcb/Thbs4* aged muscle. Immunostaining with an antibody against fibronectin shows equally strong expression in quadriceps femoris, tibialis anterior and triceps brachii isolated from 15-month-old *Sgcb* KO and *Sgcb/Thbs4* mice. At least four mice from each group were analyzed. Bar, 75 μm.

Apart from fat deposits, pronounced fibrosis is a feature of aged *Sgcb* muscle^28,29^. To explore whether loss of Thbs4 affects severity of fibrosis in β-sarcoglycan-deficient muscles, we analyzed fibrotic lesions in quadriceps, tibialis anterior and triceps isolated from 15-month-old mice (Fig. 6). As demonstrated by immunostaining with a fibronectin antibody, equally extensive fibrotic lesions were a feature of *Sgcb* and *Sgcb/Thbs4* muscles (Fig. 6).

**Figure 7:**
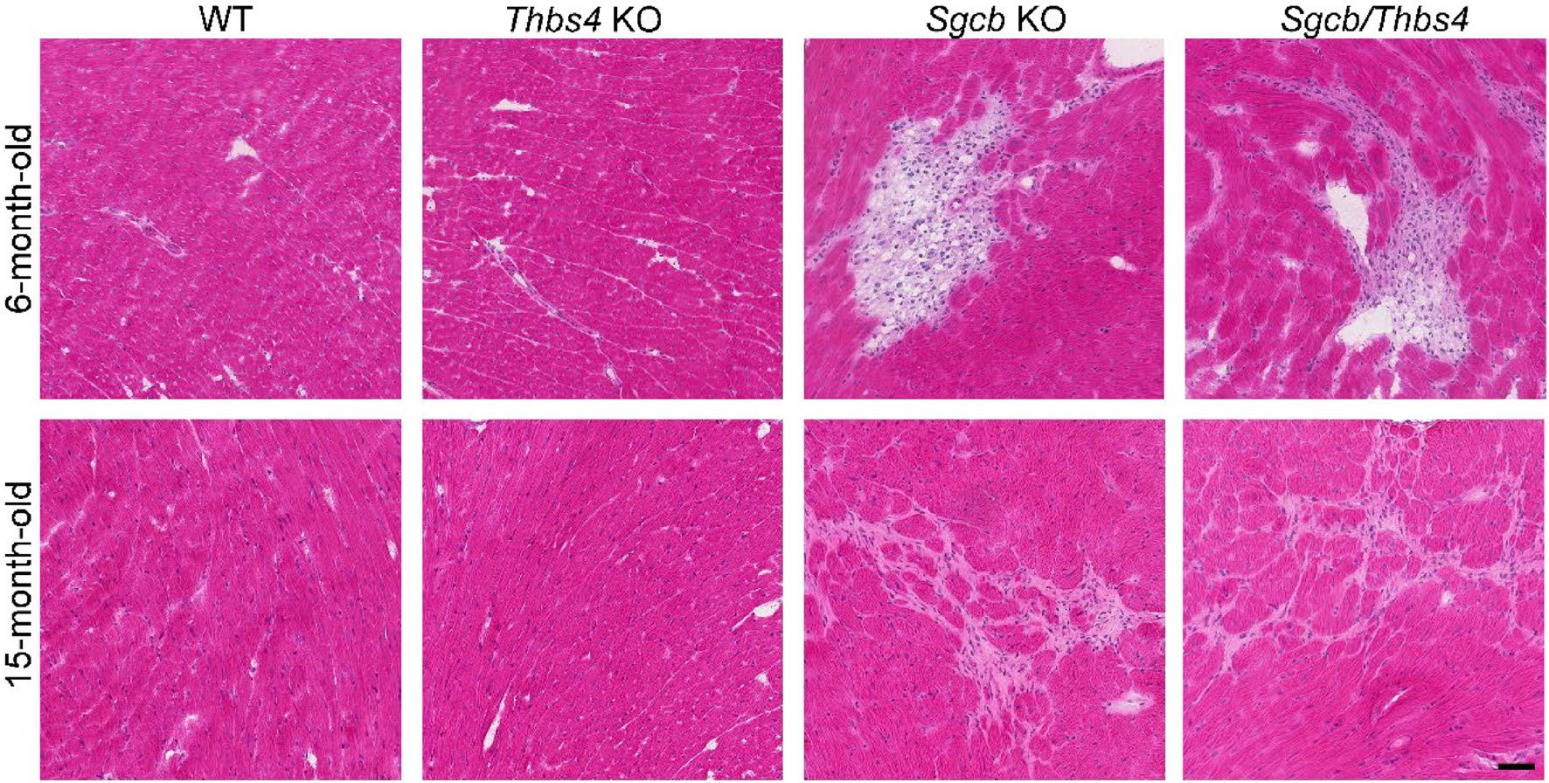
Deletion of Thbs4 does not aggravate cardiomyopathy in *Sgcb* mouse. Hematoxylin and eosin staining of heart muscle isolated from 6-month-old and 15-month-old WT, *Thbs4* KO, *Sgcb* KO and *Sgcb/Thbs4* mice. The same degree of focal necrosis (6-month-old mice) and fibrosis (15-month-old mice) was observed in *Sgcb* KO and *Sgcb/Thbs4* mice. Two mice from each group were analyzed. Bar, 50 μm.

A particularly significant role was attributed to Thbs4 in protection of cardiomyocytes upon stress response. Consequently, ablation of the Thbs4 gene enhanced cardiomyocyte membrane instability in *mdx* mice^49^. Absence of Thbs4 did not affect heart muscle in 3-month-old *Sgcb* animals, but major pathogenic features (necrosis and fibrosis) become evident at 5 months of age in *Sgcb* cardiac muscle^28^. Hence, it was highly relevant to carry out analysis of heart phenotype at later stages of the disease in *Sgcb/Thbs4* mouse. Thbs4 deletion did not cause any worsening of heart abnormalities in double knockout mice at 6 months and 15 months of age. Both *Sgcb* and *Sgcb/Thbs4* hearts displayed the same degree of necrotic lesions at 6 months and fibrotic lesions at 15 months of age (Fig. 7).

In summary, not a single dystrophic feature was aggravated by deletion of Thbs4 in *Sgcb* mice at any stage of the disease, suggesting minor involvement of the molecule in regulation of the pathophysiology in LGMD2E. This contrasts sharply with robust role of Thbs4 in modulation of the phenotype in *Sgcd* mice and *mdx* mice^43,49^.

### Deletion of Thbs4 does not exacerbate muscular dystrophy in laminin α2 chain-deficient mice

**Figure 8:**
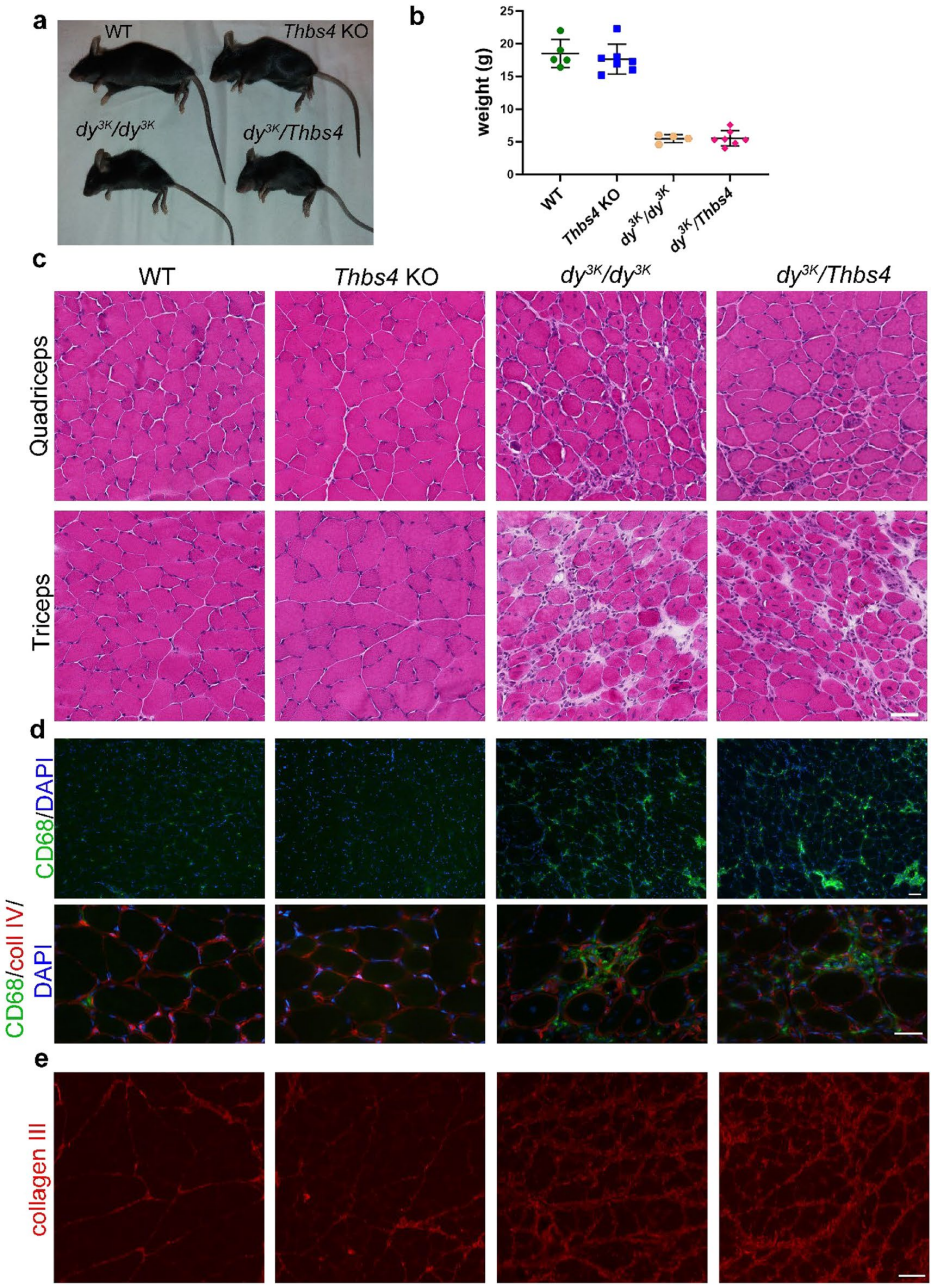
Deletion of Thbs4 does not exacerbate pathohistological features of muscular dystrophy in laminin α2-deficient muscles. (**a**) Double knockout mice (*dy*^*3K*^*/Thbs4*) present the same outward phenotype as single knockout (*dy*^*3K*^*/dy*^*3K*^) animals; severe muscle wasting, and loss of muscle mass is evident. (**b**) Body weight analysis of WT, *Thbs4* KO, *dy*^*3K*^*/dy*^*3K*^ and *dy*^*3K*^*/Thbs4* mice. There is no significant difference in body weight between *dy*^*3K*^*/dy*^*3K*^ and *dy*^*3K*^*/Thbs4* mice (p = 0.9998), but they weigh significantly less than control mice (p = 0.8401, one-way ANOVA followed by Tukey’s multiple comparison test. (**c**) Hematoxylin and eosin staining of quadriceps and triceps muscles of 5-week-old WT, *Thbs4* KO, *dy*^*3K*^*/dy*^*3K*^ and *dy*^*3K*^*/Thbs4* mice. Dystrophic muscles (*dy*^*3K*^*/dy*^*3K*^ and *dy*^*3K*^*/Thbs4*) display histopathological signs of muscular dystrophy typical for laminin α2 chain deficiency: muscle fiber atrophy, centrally located nuclei, fibrosis, infiltration of inflammatory cells. No difference in degree of dystrophic changes was observed between *dy*^*3K*^*/dy*^*3K*^ and *dy*^*3K*^*/Thbs4* muscle. (**d**) Immunofluorescence staining for CD68 (green) in quadriceps muscle of 5-week-old WT, *Thbs4* KO, *dy*^*3K*^*/dy*^*3K*^ and *dy*^*3K*^*/Thbs4* mice. CD68 depicts macrophage/monocyte infiltration. Higher magnification is shown in the bottom panel. Collagen IV (red) outlines the muscle fiber. DAPI (blue) visualizes nuclei. Dystrophic muscle (*dy*^*3K*^*/dy*^*3K*^ and *dy*^*3K*^*/Thbs4*) displays an increased number of macrophages/monocytes compared to healthy control muscles. No difference in the amount of macrophage/monocyte infiltrates was seen between *dy*^*3K*^*/dy*^*3K*^ and *dy*^*3K*^*/Thbs4*. (**e**) Immunofluorescence staining with collagen III in quadriceps muscle of 5-week-old WT, *Thbs4* KO, *dy*^*3K*^*/dy*^*3K*^ and *dy*^*3K*^*/Thbs4* mice. Increased collagen III content, which represents accumulation of fibrosis, was observed in dystrophic muscles compared to healthy muscles. No difference regarding amount of collagen III between *dy*^*3K*^*/dy*^*3K*^ and *dy*^*3K*^*/Thbs4* was visible. Bars, 50 μm.

*Dy*^*3K*^*/dy*^*3K*^ mice display outward hallmarks of muscular dystrophy at 5 weeks of age such as emaciation, decreased body size, muscle wasting and weight loss^32^ (Figs. 8a, b). Deletion of Thbs4 did not worsen the outward phenotype of *dy*^*3K*^*/dy*^*3K*^ mice: no significant differences in survival, overall size, muscle wasting, and body weight could be observed between *dy*^*3K*^*/dy*^*3K*^ and *dy*^*3K*^*/Thbs4* double knockout mice (Fig. 8a, b). Because both *dy*^*3K*^*/dy*^*3K*^ and *dy*^*3K*^*/Thbs4* animals were very sick, we could not perform any activity test.

Laminin α2-deficient muscle displays dystrophic features such as muscle fiber atrophy, centrally located nuclei, fibrosis and infiltration of inflammatory cells^25^. Both *dy*^*3K*^*/dy*^*3K*^ and *dy*^*3K*^*/Thbs4* muscle showed those dystrophic features (Fig. 8c). However, no difference in degree of dystrophic changes was observed between *dy*^*3K*^*/dy*^*3K*^ and *dy*^*3K*^*/Thbs4* muscle (Fig. 8c). Acute inflammatory response in the form of macrophage infiltration is a common characteristic of the laminin α2-deficient dystrophic muscle at the early stage of the disease^31^. Here, we investigated whether the deletion of Thbs4 would exacerbate inflammatory response in *dy*^*3K*^*/dy*^*3K*^ muscle. Staining for CD68 (macrophages/monocytes) was performed in triceps and quadriceps of WT, *Thbs4* KO, *dy*^*3K*^*/dy*^*3K*^ and *dy*^*3K*^*/Thbs4*. We found an increase of inflammatory cell infiltration between control muscles and dystrophic muscles (Fig. 8d), but no changes in the amount of macrophage/monocyte infiltrates in *dy*^*3K*^*/Thbs4* muscle compared to *dy*^*3K*^*/dy*^*3K*^ were evident (Fig. 8d).

To investigate whether deletion of Thbs4 in laminin α2 chain-deficient muscles leads to an increase of fibrosis, muscles from WT, *Thbs4* KO, *dy*^*3K*^*/dy*^*3K*^ and *dy*^*3K*^*/Thbs4* mice were stained with a collagen III antibody (Fig. 8e). Immunostaining showed increased collagen content in dystrophic muscles (*dy*^*3K*^*/dy*^*3K*^ and *dy*^*3K*^*/Thbs4*) compared with control muscles (WT, *Thbs4* KO). In healthy muscles collagen III is limited to perimysium, while dystrophic muscles display thickened deposits of collagen III in perimysium and increased collagen III expression in endomysium (Fig. 8e). We detected no difference in amounts of fibrotic lesions between *dy*^*3K*^*/dy*^*3K*^ and *dy*^*3K*^*/Thbs4* mice (Fig. 8e).

### Thbs4 is present merely in the extracellular compartment in muscles of laminin α2 chain-deficient and β-sarcoglycan-deficient mice

**Figure 9:**
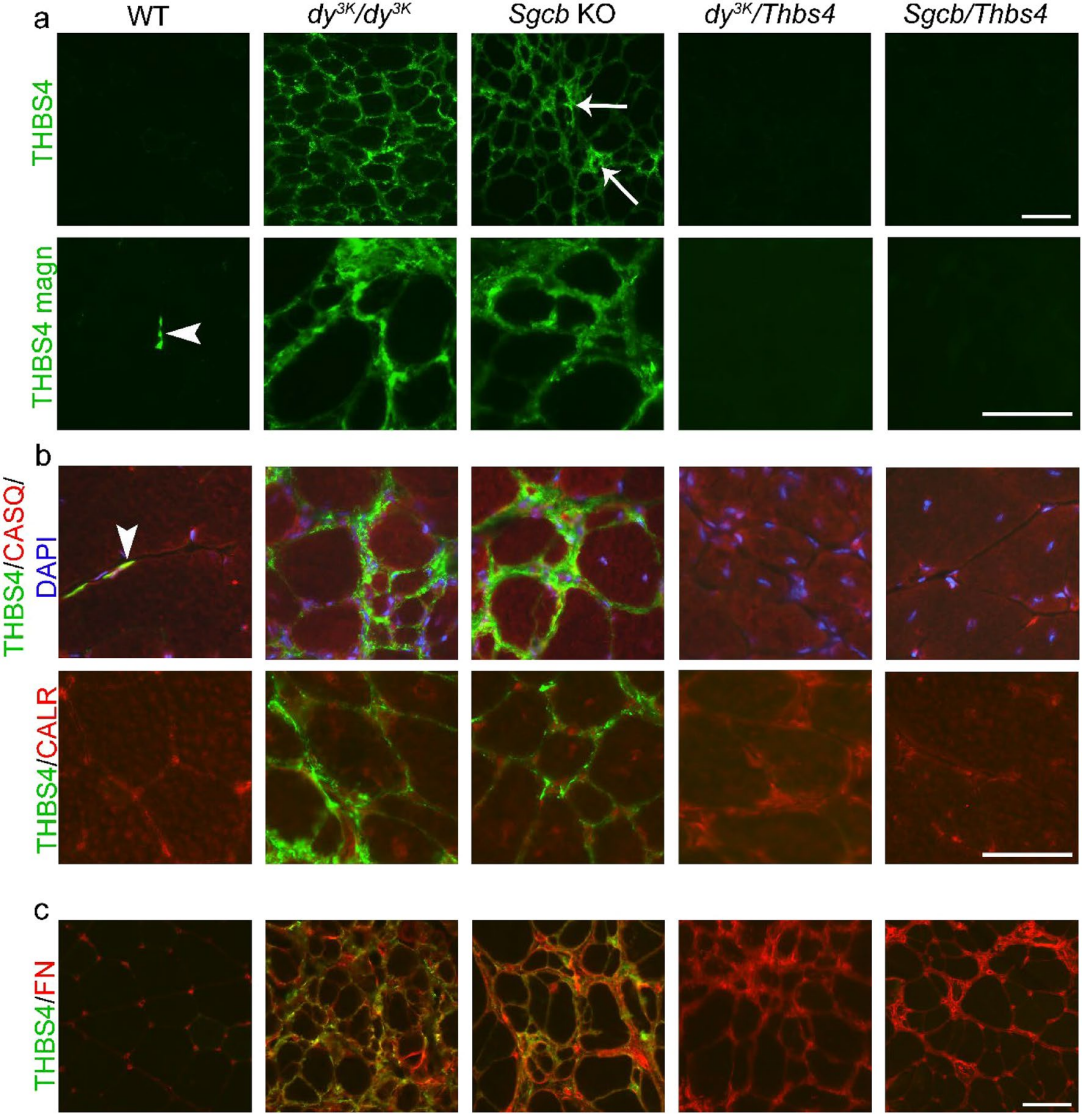
Thbs4 is upregulated merely in the extracellular compartment in laminin α2 chain-deficient and β-sarcoglycan-deficient muscles. (**a**) Immunofluorescence staining for Thbs4 (green) in quadriceps muscle of 3-month-old WT, *dy*^*3K*^*/dy*^*3K*^, *Sgcb* KO, *dy*^*3K*^*/Thbs4* and *Sgcb/Thbs4* mice at low and high magnification. To simplify the figure, *Thbs4* KO muscle was omitted, Thbs4 deletion is represented by respective double knockout muscles. Thbs4 is expressed at low levels in WT muscle, but strongly expressed in the neuromuscular junction (white arrowhead, also in panel b). As expected, Thbs4 is not expressed in *Sgcb/Thbs4* and *dy*^*3K*^*/Thbs4* muscle. An upregulation of Thbs4 was evident in *dy*^*3K*^*/dy*^*3K*^ muscle and *Sgcb* KO muscle. White arrows indicate high Thbs4 expression at the dystrophic areas in *Sgcb* KO muscle. High magnification of Thbs4 staining presents extensive deposits of the protein around the cells, but not intracellularly. (**b**) Double immunofluorescence staining for Thbs4 (green) and calsequestrin (red) (top panel) and Thbs4 (green) and calreticulin (red) (bottom panel). Thbs4 is absent from sarcoplasmic reticulum in *dy*^*3K*^*/dy*^*3K*^ and *Sgcb* KO muscles. (**c**) Double immunofluorescence staining of Thbs4 (green) and fibronectin (red). Thbs4 colocalizes with fibronectin in the extracellular compartment in *dy*^*3K*^*/dy*^*3K*^ and *Sgcb* KO muscles. At least three mice from each group were analyzed. Bars, 75 μm.

It has previously been demonstrated that Thbs4 is upregulated in δ-sarcoglycan-deficient and dystrophin-deficient muscle^43^. Microarray and proteomic analyses have shown an upregulation of Thbs4 in laminin α2-deficient muscle^53,54^. We performed qPCR to analyze Thbs4 transcripts in *dy*^*3K*^*/dy*^*3K*^ and *Sgcb* muscles. A trend for upregulation of Tbhs4 mRNA in both dystrophic groups was observed (Supplementary Fig. S5). An immunofluorescence staining with antibody against Thbs4 was performed to determine protein expression and localization in *Sgcb* and *dy*^*3K*^*/dy*^*3K*^ muscle. Thbs4 was expressed at very low levels in WT muscle, but it was present in neuromuscular junctions^55^ (Fig. 9a, arrowheads). As expected, Thbs4 was not expressed in *Thbs4* KO (data not shown), *Sgcb/Thbs4*, or *dy*^*3K*^*/Thbs4* muscles (Fig. 9a). In agreement with previous findings, we detected extensive upregulation of Thbs4 in *dy*^*3K*^*/dy*^*3K*^ muscle (Fig. 9a). Strongly increased production of Thbs4 was also found in *Sgcb* KO muscle (Fig. 9a). Thbs4 was expressed at high levels especially at dystrophic areas (Fig. 9a, white arrows). This indicates that Thbs4 is secreted in connection with inflammation, muscle remodeling and regeneration^56^. High magnification images indicated no clear intracellular deposits of Thbs4 (Fig. 9a).

Further, we analyzed in detail the localization of Thbs4 staining. It has been shown before that Thbs4 is located intracellularly in muscles of δ-sarcoglycan-deficient mice, from where it induces an ER (endoplasmic reticulum) stress response, which promotes trafficking of stabilizing protein complexes to the sarcolemma^43^. To investigate whether Thbs4 is located in the sarcoplasmic reticulum (SR) in *Sgcb* KO and *dy*^*3K*^*/dy*^*3K*^ muscle, we performed a double immunofluorescence staining of Thbs4 and calsequestrin, as well as Thbs4 and calreticulin (Fig. 9b). Calreticulin is expressed in the ER, including the SR, whereas calsequestrin is SR-specific. We have not detected a clear signal for Thbs4 in the SR (Fig. 9b).

Extensive production and deposition of extracellular matrix proteins is a hallmark of muscular dystrophies. We stained for fibronectin, a glycoprotein expressed in the extracellular space upon tissue remodeling and in fibrotic lesions. Combined staining of Thbs4 and fibronectin indicated an extracellular localization of Thbs4, both in *dy*^*3K*^*/dy*^*3K*^ and *Sgcb* KO muscle (Fig. 9c). Together, these results point towards Thbs4 role in tissue remodeling and its contribution to increased pool of extracellular proteins. This further indicates that Thbs4 might not be involved in sarcolemma-stabilizing mechanisms and increased protein trafficking in *Sgcb* and *dy*^*3K*^*/dy*^*3K*^ muscle, as these mechanisms seem to require substantial levels of Thbs4 in the SR. ^43,49,56^.

### Sarcolemmal protein complexes are not reduced in laminin α2-deficient or β-sarcoglycan-deficient mice with Thbs4 deletion

To further substantiate the results shown in Fig. 9, we investigated trafficking of adhesion complexes, we focused on expression of dystroglycans and integrins. As previously demonstrated, Thbs4 increases trafficking of stabilizing protein complexes to the sarcolemma in δ-sarcoglycan-deficient muscles, for example the DGC and integrins^43^. Conversely, deletion of Thbs4 in wild-type mice reduced sarcolemmal levels of integrin α7 and integrin β1^43^.

**Figure 10:**
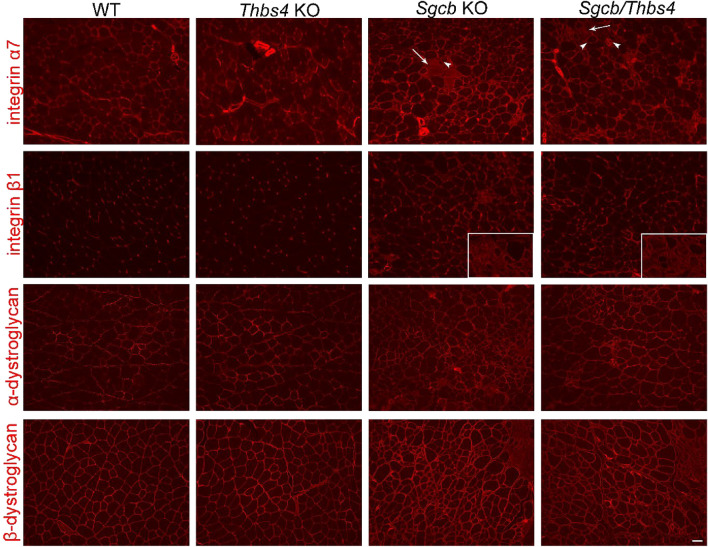
Expression pattern of sarcolemmal receptor complexes is similar in *Sgcb and Sgcb/Thbs4* muscle. Immunofluorescence staining for integrin α7, integrin β1, α-dystroglycan and β-dystroglycan in quadriceps muscle of WT, *Thbs4* KO, *Sgcb* KO, and *Sgcb/Thbs4* mice (2–3-month-old animals). Integrin α7 is upregulated in dystrophic groups compared to controls, but no difference can be seen between *Sgcb* KO and *Sgcb/Thbs4* muscle. Loss of integrin expression has been observed at the sarcolemma in necrotic cells (white arrows), while it was maintained in living cells in dystrophic areas (white arrowheads). No reduction of integrin α7 is seen in *Thbs4* KO muscle compared to WT muscle. The same staining pattern was detected for integrin β1. Insets show necrotic areas, where expression of integrin β1 was clearly preserved in living cells. Expression of α- and β-dystroglycan was preserved in *Sgcb* KO and *Sgcb/Thbs4* mice. No visible change in expression between dystrophic and healthy groups was observed.

To assess how deletion of Thbs4 affects protein complexes stabilizing sarcolemma in β-sarcoglycan-deficient muscle, we performed an immunostaining for the integrins α7, β1 and dystroglycans in *Sgcb* KO, *Sgcb/Thbs4*, *Thbs4* KO and WT muscle (quadriceps and tibialis anterior). Immunostaining showed an upregulation of integrin α7 in dystrophic muscles, with no apparent difference between *Sgcb* KO and *Sgcb/Thbs4* muscle (quadriceps, Fig. 10 top row; tibialis anterior, Supplementary Fig. S4). Expression of integrin α7 was lost in necrotic muscle fibers (white arrows). We detected a similar expression pattern for integrin β1 (Fig. 10). It is noteworthy that living muscle cells expressed integrins at the cell surface even within necrotic areas (Fig. 10, white arrowheads for integrin α7 and inset for integrin β1). No difference in levels of integrin α7 or integrin β1 could be observed between WT and *Thbs4* KO (Fig. 10). Immunofluorescence with antibodies against α- and β-dystroglycan showed no visible reduction of dystroglycans levels in *Sgcb/Thbs4* muscle compared to *Sgcb* muscle (Fig. 10). These results indicate no disruption of protein trafficking to sarcolemma upon Thbs4 deletion. Immunostaining is not the optimal method to determine whether adhesion complexes are membrane anchored. It has been shown that sarcolemma-bound portion of α-dystroglycan is severely reduced in sarcoglycan deficiencies despite the positive immunofluorescence signal in the proximity of cell membrane^28^. It remains to be determined whether integrins are incorporated at the sarcolemma in *Sgcb/Thbs4* muscles. However, the staining for both integrins and dystroglycans demonstrate clearly that the receptors were directed to the sarcolemmal compartment.

**Figure 11:**
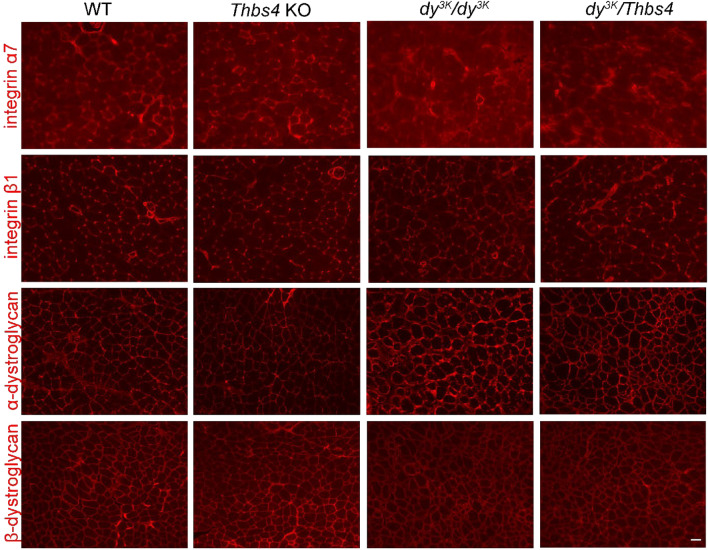
Expression pattern of sarcolemmal receptor complexes is similar in *dy*^*3K*^*/dy*^*3K*^ and *dy*^*3K*^*/Thbs4* muscle. Immunostaining for integrin α7, integrin β1, α- and β-dystroglycan in quadriceps of WT, *Thbs4* KO, *dy*^*3K*^*/dy*^*3K*^, and *dy*^*3K*^*/Thbs4* mice. Integrin α7 is severely reduced in *dy*^*3K*^*/dy*^*3K*^, and *dy*^*3K*^*/Thbs4* muscle, whereas integrin β1 is expressed in similar manner as in control mice. Dystroglycan levels are not reduced in *Thbs4* KO and *dy*^*3K*^*/Thbs4* muscle. Bars, 50 μm. At least three mice from each group were analyzed.

In order to assess how deletion of Thbs4 affects sarcolemmal complexes inlaminin α2-deficiency, immunostaining for α-dystroglycan, β-dystroglycan and integrin α7β1 was performed in *dy*^*3K*^*/dy*^*3K*^, *dy*^*3K*^*/Thbs4*, *Thbs4* KO, and WT muscle. The expression pattern of integrin α7 and β1 was also similar in *dy*^*3K*^*/dy*^*3K*^ and *dy*^*3K*^*/Thbs4* muscle: integrin α7 was severely reduced and integrin β1 was maintained in both mutants (Fig. 11). Signal for α-dystroglycan appeared stronger in muscles from both dystrophic groups. However, immunostaining did not indicate a downregulation of neither α- nor β-dystroglycan in *dy*^*3K*^*/Thbs4* muscle compared to *dy*^*3K*^*/dy*^*3K*^ muscle (Fig. 11). This further suggests that Thbs4 does not have an intracellular role in promoting receptor trafficking to the sarcolemma in laminin α2-deficient muscular dystrophy. In summary, even if there are subtle differences in the expression levels of adhesion receptors between single and double knockouts (*Sgcb* vs *Sgcb/Thbs4* and *dy*^*3K*^*/dy*^*3K*^ vs *dy*^*3K*^*/Thbs4*), clearly, they do not make difference for the phenotype.

## Discussion

Thrombospondin-4 has emerged to be a promising therapy target for both limb-girdle muscular dystrophy type 2F and Duchenne muscular dystrophy, as its overexpression mitigated dystrophic phenotype in mouse models of these muscular dystrophies. It was shown that the improved phenotype arose from increased stabilization of the sarcolemma by enhancing transport of sarcolemmal protein complexes in these two muscular dystrophies in mouse models^43^. It has therefore been proposed that Thbs4 could be a therapeutic agent for all muscular dystrophies involving adhesion complexes in the sarcolemma.

In this study, we aimed to verify whether Thbs4 is a universal target for DGC-related muscular dystrophies. We explored Thbs4 deletion in mouse models of β-sarcoglycan deficiency and laminin α2 chain deficiency. The deletion of the Thbs4 gene did not result in an exacerbation of muscular dystrophy in any of these two disorders, indicating that Thbs4 does not have the same disease-modifying effect as has been shown in mice models of Duchenne muscular dystrophy and LGMD2F. This was demonstrated by analysis of outward phenotype, muscle function, and pathohistological features at wide range of ages.

A possible explanation for the absence of disease-modifying effect of Thbs4 in *Sgcb* mice is that Thbs4 is merely located in the extracellular compartment in *Sgcb* muscle, and distinct intracellular portion of Thbs4 was not detected. This contrasts with *Sgcd* muscle, where Thbs4 is expressed intracellularly, inducing an ER stress response that increases trafficking of stabilizing protein complexes to the sarcolemma^43^. Consequently, the expression of adhesion complexes (integrin α7β1 and dystroglycans) was not further perturbed by deletion of Thbs4 in *Sgcb* muscle, as it was seen in *Sgcd* mice^43^. Moreover, the number of EBD-positive cells remained unaltered after Thbs4 deletion, thus indicating that Thbs4 does not affect membrane stability in muscles of *Sgcb* mice. Importantly, the extracellular deposits of Thbs4 were allocated to dystrophic/necrotic areas of muscle, which points out the role of Thbs4 in repair mechanism and tissue remodeling^57–60^. However, it is not excluded that Thbs4 presence in the extracellular milieu could also be a sign of generally increased production of extracellular matrix proteins that later contribute to fibrosis in muscle disease. In dystrophic muscle, representing a highly disorganized milieu, the remodeling function could turn into fibrotic process. For example, it has been demonstrated that thrombospondin-4 promotes TGF-β signaling and contributes to fibrosis in hypertrophic scar formation^61^. On the other hand, anti-fibrotic properties of Thbs4 have been described and deletion of Thbs4 has been shown to promote fibrosis and inflammation in heart and vessels^57,58^. Yet, we have not detected enhancement of these processes in *Sgcb* KO and *dy*^*3K*^*/dy*^*3K*^ muscles lacking Thbs4.

The results described in this study are somewhat surprising because pathogenesis of β-sarcoglycan-deficient and δ-sarcoglycan-deficient muscular dystrophy is similar. Deficiency of either of these sarcoglycan subtypes leads to loss of the DGC and thus instable sarcolemma^28,34–37,62^. It would therefore be logical to assume that loss of Thbs4 in *Sgcb* mice would lead to an exacerbation of muscular dystrophy in the same way as was seen in *Sgcd* model. It is possible that deletion of Thbs4 affects β-sarcoglycan-deficient and δ-sarcoglycan-deficient muscles differently due to individual properties of these two proteins. It has previously been shown that each of the sarcoglyans has unique characteristics, for example specific binding sites, and might perform unique functions^12^. δ-sarcoglycan has been shown to interact directly with a H^+^-ATPase^63^ and α-sarcoglycan acts as an ATPase^64,65^. Comprehensive roles and interactions of each of the sarcoglycans, including β-sarcoglycan, have not been fully understood. Hence, Thbs4 deletion in two different sarcoglycan knockout models could have diverse repercussions.

Finally, the precise roles of thrombospondins in different pathologies have not yet been clearly delineated. The thrombospondin family have been shown to scaffold cell–matrix and cell–cell interactions, regulate the molecular composition of the extracellular matrix in response to tissue injury and tissue healing^46–48,60,66,67^. Both protective and detrimental effects have been associated with expression of thrombospondins in various cell types and at specific stages after injury^43,46,49,57,58,68^. In this context, the role of thrombospondin-4 in pathogenesis of various diseases might be regulated in a very fine-tuned way due to subtle differences in molecular signature of pathologic milieu.

It is also worth mentioning that we did not observe any pathological changes in muscles from single knockout mice lacking Thbs4 even at age of 15 months. This finding is discrepant to data published by Vanhoutte et al., where Thbs4 knockout mice showed signs of mild muscular dystrophy at 6 months of age^43^. We have not investigated red skeletal muscle with high oxidative metabolism (soleus), in which decreased muscle mass has been observed^48^, but we have not detected strength deficit nor clear weight reduction in *Thbs4* KO mice. Lack of dystrophic phenotype in Thbs4 knockout mice could indicate why we did not observe worsening of dystrophic symptoms in *Sgcb/Thbs4* animals. One must also mention that our study and the study by Vanhoutte et al. differ substantially in experimental design and experimental protocols. Additionally, *Thbs4* KO mice were not backcrossed to *Sgcb* background. This could give rise to differences in results obtained.

Thbs4 is clearly upregulated in mice with laminin α2-deficiency, as has been demonstrated here and previously by others^53,54^. However, just as in *Sgcb* mice, Thbs4 was found only in the extracellular compartment in laminin α2-deficient muscle, and we did not observed exacerbation of muscular dystrophy by deletion of Thbs4. This further supports our theory that Thbs4 does not have protective role in muscle when located extracellularly, at least in the two investigated mouse models. Membrane instability is not considered to be the main reason for cell degeneration and muscular dystrophy in laminin α2 deficiency. Instead, it is believed that lack of signaling between laminin α2 and transmembrane receptors integrin α7β1 and dystroglycan leads to myofiber degeneration^40^. Since the phenotype of double knockout was not worsened, it is safe to assume that signaling events in double knockout muscles were no further perturbed. In agreement with this, we did not detect a difference in expression of dystroglycan in double knockout mice compared to *dy*^*3K*^*/dy*^*3K*^ mice. This confirms that Thbs4 does not have an obvious intracellular role in laminin α2-deficiency. It is also noteworthy that the expression pattern of Thbs4 in β-sarcoglycan deficiency and laminin α2 chain deficiency is comparable even though they do not share all pathological mechanisms and clinical manifestations. In contrast, it remains to be clarified why this expression pattern differs so much between the two much more similar diseases: LGMD2E and 2F.

Taken together, our findings suggest that Thbs4 might not have the same disease-protective effect in mouse models of LGMD2E and LAMA2-RD as it was shown to have in mouse models of Duchenne muscular dystrophy and LGMD2F. We suggest that the differences stem from the fact that Thbs4 is present only in the extracellular space in muscles of *Sgcb* and *dy*^*3K*^*/dy*^*3K*^ mice, and consequently does not induce intracellular trafficking of protein complexes to stabilize the sarcolemma. We cannot exclude that overexpression of Thbs4 in *Sgcb* and *dy*^*3K*^*/dy*^*3K*^ mice by transgenic means and strong expression of Thbs4 in the sarcoplasmic reticulum could result in improvement of the phenotype. On the other hand, it could also contribute to an additional increase of extracellular matrix content around muscle fibers that is detrimental for muscle function. In any case, lack of this evidence is clearly a limitation of our study. Nevertheless, we suggest that Thbs4 might not necessarily be a universal therapeutic target for muscular dystrophies involving the DGC. These findings point out the importance of verification of promising therapeutic approaches, even though it would be logically justified to assume that the same therapeutic strategy would be effective in diseases with similar pathogenesis. Our study also demonstrates how vastly complicated muscular disorders are, when one protein plays different roles in two closely related disorders as LGMD2F and LGMD2E. Consequently, molecular pathology of similar diseases could be surprisingly unique and should be thoroughly compared.

## Materials and methods

### Ethics statement

All animal experiments were approved by the Malmö/Lund (Sweden) Ethical Committee for Animal Research (ethical permit numbers M152-14 and M173-19), in accordance with guidelines from the Swedish Board of Agriculture. All experiments were performed in accordance with laws and regulations issued by the Swedish Board of Agriculture. Experiments involving live animals were reported according to the ARRIVE guideline.

### Animal models

Laminin α2-deficient *dy*^*3K*^*/dy*^*3K*^ mice were previously described^32^ and maintained in the animal facilities of Biomedical Center (Lund, Sweden). Mice carrying null mutations in the β-sarcoglycan gene and the thrombospondin-4 gene, respectively were purchased from the Jackson Laboratory (B6.129-Sgcbtm1Kcam/1J, B6.129P2-Thbs4tm1Dgen/J)^28,69^ and bred in-house. Heterozygous *dy*^*3K*^/+ mice and *Thbs4* KO mice were crossed. Their heterozygous progenies were bred to create *dy*^*3K*^*/Thbs4* double knockout, *Thbs4* KO, *dy*^*3K*^*/dy*^*3K*^, and control mice (wild-type, *dy*^*3K*^/+, *Thbs4*/+ or *dy*^*3K*^/+; *Thbs4*/+). Also, *Sgcb* heterozygous mice and *Thbs4* KO mice were crossed, and their heterozygous progenies were bred to create *Sgcb/Thbs4* double knockout, *Sgcb* KO, *Thbs4* KO and control mice (wild-type, *Sgcb*/+, *Thbs4*/+ or *Sgcb*/+; *Thbs4*/+). Animals were genotyped according to standard procedures^70^ and protocols from the Jackson Laboratory.

### Tissue preparation

*Sgcb/Thbs4*, *Sgcb* KO, *Thbs4* KO and wild-type (WT) mice were sacrificed at 2, 3, 6 and 15 months of age. *Dy*^*3K*^*/Thbs4* and *dy*^*3K*^*/dy*^*3K*^ mice were sacrificed at 5 weeks of age along with their age-matched healthy controls and *Thbs4* KO controls. Skeletal muscles (quadriceps femoris, triceps brachii, tibialis anterior, diaphragm) and heart muscle from WT, *Sgcb* KO, *Sgcb/Thbs4*, *Thbs4* KO, *dy*^*3K*^*/dy*^*3K*^, and *dy*^*3K*^*/Thbs4* were isolated after euthanasia with CO_2_ asphyxiation. Tissues were embedded in O.C.T. compound (Tissue-Tek^®^ O.C.T; Sakura Finetek) and frozen in liquid nitrogen.

### Histological analysis

Transverse cryosections of 7 µm were stained with hematoxylin and eosin (H&E) to assess muscle morphology. Briefly, sections were incubated in Harris hematoxylin (Histolab, Göteborg, Sweden) (1 min), water, 0.5% acid alcohol, water, Scott’s tap water, water, 80% ethanol, eosin (30 s) (Histolab, Göteborg, Sweden), 95% and 100% ethanol, and xylene, and mounted in Pertex (Histolab, Göteborg, Sweden). Fifteen-month-old muscles were stained with Oil red O to visualize fat. Briefly, sections were fixed in formaldehyde-PBS, washed in water and 60% isopropanol, then incubated in Oil red O (20 min), washed in water and 60% isopropanol, incubated in Hematoxylin (10 min), washed in water, and mounted in water-soluble mounting-media.

### Immunohistochemistry

Cryosections were fixed in ice-cold acetone for 8 min at −20 °C and then washed in PBS. For α-dystroglycan staining sections were fixed in 8% formaldehyde and ice-cold methanol. Sections were blocked in 3% BSA-PBS for 20 min and incubated for 90 min at room temperature with following primary antibodies diluted in blocking buffer: anti-Thbs4: sheep anti-mouse, R&D Systems, cat# AF7860, 1:200; anti-fibronectin: rabbit anti-human, Abcam, cat#23750, 1:500; anti-calreticulin: rabbit anti-mouse, Abcam, cat# ab2907, 1:100; anti-calsequestrin; rabbit anti-mouse, Invitrogen, cat# PA1-913, 1:200; anti-integrin α7^71^: rabbit anti-mouse, 1:100; Integrin β1: rat anti-mouse CD29, BD Pharmingen, cat# 550531, 1:50; anti-collagen III: goat anti-human, Southern Biotech, cat# 1330-01, 1:100; anti-CD68: rat anti-mouse monoclonal (clone FA-11), Thermofisher Scientific, cat# 14-0681-82, 1:200; anti-collagen IV: rabbit anti-mouse, Millipore, cat# AB756P, 1:200; mouse monoclonal anti-α-dystroglycan (clone IIH6), Millipore, cat# 05-593, 1:50; rabbit anti-β-dystroglycan^70^, 1:50; anti laminin: rabbit anti mouse, Abcam, cat# ab11575, 1:500; donkey anti-mouse IgG-546 (conjugated with 546 fluorochrome); Invitrogen; cat# A11003, 1:500. After washing with PBS, appropriate secondary antibodies (except for IgG staining) diluted 1:500 in PBS were applied for 1 h at room temperature, and then for 2 min with DAPI nuclear DNA stain with PBS washing before and after. Sections were mounted in Invitrogen PermaLong Diamond Antifade Mountant (Thermo Fisher Scientific) and analyzed using a Zeiss Axioplan 2 microscope. The images were captured using ORCA camera and Openlab software (version 4). The same exposure times were used when photographing the same staining in different mouse groups.

### Functional tests

*Sgcb/Thbs4*, *Sgcb* KO, *Thbs4* KO and wild-type mice were subjected to functional tests at 2 months and 3 months of age. Open field activity test: mice were transferred to a new cage for 5 minutes. Time spent on active exploration of the cage was measured. Stand up test: during the open field activity test, the number of times that the animal was rising to a standing position on hind legs were noted. Grip strength test: the grip strength was measured by letting animals pull a metal bar with their forelimbs on a grip strength meter (Columbus Instruments). Animals were subjected to the experiment five times, of which the three highest scores were considered, and the mean value was calculated. Body weight was recorded in parallel. Results are presented as normalized strength (force (g)/body weight (g)). Forced treadmill running: two-month-old *Sgcb/Thbs4*, *Sgcb* KO, *Thbs4* KO and wild-type mice were exercised on a treadmill Exer 6 M (Columbus Instruments) at a downhill angle of 15°. Mice were exercised at a speed of 6 m/min for 2 min and then increasing the speed to 13 m/min until fatigue or for a maximum of 30 min.

### Evans blue dye uptake

To assess sarcolemmal capacity of skeletal muscle cells, mice were injected intraperitoneally with Evans blue dye (EBD) dissolved in sterile saline (concentration: 0.5 mg EBD/0.05 ml saline; amount injected: 50 µL/10 g body weight). After 24 h, animals were sacrificed, and muscles were collected and frozen in liquid nitrogen. Muscle cryosections were stained with laminin antibody to visualize EBD-positive and EBD-negative muscle fibers. Pictures covering the whole muscle were taken in fluorescent microscope (10× magnification) and subsequently stitched in Photoshop (version 10.0.19041.3636, https://adobe.com). The percentage of EBD-positive fibers was then calculated using ImageJ software (version 1.53e, https://imagej.net/ij/) with cell counter plug-in.

### Necrosis quantification

Muscle cryosections (quadriceps femoris and tibialis anterior) from 3-month-old mice were stained with anti-mouse IgG conjugated with 546 fluorochrome. Pictures covering the whole muscle were taken (10× magnification) and stitched in Photoshop (version 10.0.19041.3636, https://adobe.com). In order to avoid false-positive signal for quantification, artefacts, non-muscle tissue and background staining were manually removed from images using Photoshop (version 10.0.19041.3636, adobe.com). No other modifications were applied to images. The area corresponding to IgG labelling was quantified in relation to the entire area of muscle cross-section using ImageJ software (version 1.53e, https://imagej.net/ij/).

### mRNA analysis

RNA was isolated from 2-month-old wild-type, *Sgcb* and *Thbs4* KO quadriceps muscle and from 5-week-old *dy*^*3K*^*/dy*^*3K*^ quadriceps muscle using Qiagen RNeasy Plus Universal Kit (Qiagen) according to manufacturer’s specification. Briefly: muscles were incubated in Qiazol lysis reagent and homogenized in TissueLyser (Qiagen). Genomic DNA Eliminator Solution was added, followed by chloroform. Samples were centrifuged at 4 °C in order to separate RNA from other cellular components. The aqueous phase containing RNA was transferred to RNA binding columns and washed in several steps. RNA was eluted with RNase free water by brief centrifugation. RNA concentration was determined using Nanodrop (Thermofisher). The quality of RNA samples was assessed using Agilent 2100 Bioanalyzer (Agilent RNA 6000 Nano Kit). Five hundred nanograms of muscle RNA were used to synthesize cDNA with High-Capacity cDNA Reverse Transcription Kit (Applied Biosystems) according to manufacturer’s protocol. Briefly, 10 μL RNA was transferred to a master mix containing 3.2 μL nuclease-free water, 2.0 μL 10X RT buffer, 0.8 μL 25 dNTP Mix, 2.0 μL RT random primers, 1.0 μL MultiScribe Reverse Transcriptase, 1.0 μL RNase inhibitor. Samples were placed in a thermal cycler for cDNA reverse transcription (25 °C for 10 min, 37 °C for 120 min, 85 °C for 5 min, 4 °C).

Five nanograms of cDNA was used for qPCR, together with Taqman Fast Advanced Master Mix (Applied Biosystems) and TaqMan probes detecting mouse thrombospondin-4 and β-actin (reference gene) (Assay ID: Mm00449057_m1 and Mm04394036_g1, respectively; Applied Biosystems). Each probe spanned two exons of the respective gene. The amplification was performed in a One-Step Real-Time PCR System (Applied Biosystems), including no-RT treated RNA samples as control reactions. Comparative C_T_ method was used for relative quantitation.

### Statistical analysis

All statistical analyses were performed with Graph-Pad Prism software (version 8, https://www.graphpad.com/). Averaged data were reported as mean ± SD. The number of samples is indicated on the graphs. For assessing differences between two groups student’s t-test was applied. One-way ANOVA followed by Tukey's multiple comparison post-hoc test was used when comparing more than two groups. Statistical significance was accepted for p < 0.05.

### Supplementary Information

Supplementary Figures.

## Data Availability

The datasets generated and analyzed during the current study are available from the corresponding author on reasonable request.

## References

[1] Mercuri E, Bonnemann CG, Muntoni F (2019). Muscular dystrophies. Lancet.

[2] Gawlik KI (2018). At the crossroads of clinical and preclinical research for muscular dystrophy—Are we closer to effective treatment for patients?. Int. J. Mol. Sci..

[3] Hoy SM (2023). Delandistrogene moxeparvovec: First approval. Drugs.

[4] Carmignac V, Durbeej M (2012). Cell-matrix interactions in muscle disease. J. Pathol..

[5] Wilson DGS, Tinker A, Iskratsch T (2022). The role of the dystrophin glycoprotein complex in muscle cell mechanotransduction. Commun. Biol..

[6] Talts JF, Andac Z, Gohring W, Brancaccio A, Timpl R (1999). Binding of the G domains of laminin alpha1 and alpha2 chains and perlecan to heparin, sulfatides, alpha-dystroglycan and several extracellular matrix proteins. EMBO J..

[7] Sciandra F, Gawlik KI, Brancaccio A, Durbeej M (2007). Dystroglycan: A possible mediator for reducing congenital muscular dystrophy?. Trends Biotechnol..

[8] Vainzof M, Souza LS, Gurgel-Giannetti J, Zatz M (2021). Sarcoglycanopathies: An update. Neuromuscul. Disord. NMD.

[9] Kirschner J, Lochmuller H (2011). Sarcoglycanopathies. Handb. Clin. Neurol..

[10] Guglieri M (2008). Clinical, molecular, and protein correlations in a large sample of genetically diagnosed Italian limb girdle muscular dystrophy patients. Hum. Mutat..

[11] Semplicini C (2015). Clinical and genetic spectrum in limb-girdle muscular dystrophy type 2E. Neurology.

[12] Sandona D, Betto R (2009). Sarcoglycanopathies: Molecular pathogenesis and therapeutic prospects. Expert Rev. Mol. Med..

[13] Politano L (2001). Evaluation of cardiac and respiratory involvement in sarcoglycanopathies. Neuromuscul. Disord. NMD.

[14] Zambon AA, Muntoni F (2021). Congenital muscular dystrophies: What is new?. Neuromuscul. Disord. NMD.

[15] Sarkozy A, Foley AR, Zambon AA, Bonnemann CG, Muntoni F (2020). LAMA2-related dystrophies: Clinical phenotypes, disease biomarkers, and clinical trial readiness. Front. Mol. Neurosci..

[16] Lake NJ (2023). Estimating the prevalence of LAMA2 congenital muscular dystrophy using population genetic databases. J. Neuromuscul. Dis..

[17] Ge L (2019). Congenital muscular dystrophies in China. Clin. Genet..

[18] Voit, T. & Tome, F. M. S. *The Congenital Muscular Dystrophies*. Vol. 2. 1203–1238 (McGraw-Hill, 2004).

[19] Bönnemann, C. G. & Voermans, N. C. *ECM-Related Myopathies and Muscular Dystrophies*. Vol. 2. 979–994 (Academic Press, 2012).

[20] Oliveira, J., Parente Freixo, J., Santos, M. & Coelho, T. *GeneReviews*((R)) (eds. Adam, M. P. *et al.*) (1993).

[21] Zambon AA (2020). LAMA2-related muscular dystrophy: Natural history of a large pediatric cohort. Ann. Clin. Transl. Neurol..

[22] Tan D (2021). Natural history and genetic study of LAMA2-related muscular dystrophy in a large Chinese cohort. Orphanet. J. Rare Dis..

[23] Durbeej M, Campbell KP (2002). Muscular dystrophies involving the dystrophin-glycoprotein complex: An overview of current mouse models. Curr. Opin. Genet. Dev..

[24] Ng R (2012). Animal models of muscular dystrophy. Prog. Mol. Biol. Transl. Sci..

[25] Gawlik KI, Durbeej M (2020). A family of laminin alpha2 chain-deficient mouse mutants: Advancing the research on LAMA2-CMD. Front. Mol. Neurosci..

[26] van Putten M (2020). Mouse models for muscular dystrophies: An overview. Dis. Model Mech..

[27] Araishi K (1999). Loss of the sarcoglycan complex and sarcospan leads to muscular dystrophy in beta-sarcoglycan-deficient mice. Hum. Mol. Genet..

[28] Durbeej M (2000). Disruption of the beta-sarcoglycan gene reveals pathogenetic complexity of limb-girdle muscular dystrophy type 2E. Mol. Cell.

[29] Pozsgai ER, Griffin DA, Heller KN, Mendell JR, Rodino-Klapac LR (2016). Beta-sarcoglycan gene transfer decreases fibrosis and restores force in LGMD2E mice. Gene Ther..

[30] Gawlik KI, Holmberg J, Durbeej M (2014). Loss of dystrophin and beta-sarcoglycan significantly exacerbates the phenotype of laminin alpha2 chain-deficient animals. Am. J. Pathol..

[31] Gawlik KI, Korner Z, Oliveira BM, Durbeej M (2019). Early skeletal muscle pathology and disease progress in the dy(3K)/dy(3K) mouse model of congenital muscular dystrophy with laminin alpha2 chain-deficiency. Sci. Rep..

[32] Miyagoe Y (1997). Laminin alpha2 chain-null mutant mice by targeted disruption of the Lama2 gene: A new model of merosin (laminin 2)-deficient congenital muscular dystrophy. FEBS Lett..

[33] Carmignac V, Quere R, Durbeej M (2011). Proteasome inhibition improves the muscle of laminin alpha2 chain-deficient mice. Hum. Mol. Genet..

[34] Straub V, Rafael JA, Chamberlain JS, Campbell KP (1997). Animal models for muscular dystrophy show different patterns of sarcolemmal disruption. J. Cell Biol..

[35] Ervasti JM, Campbell KP (1993). A role for the dystrophin-glycoprotein complex as a transmembrane linker between laminin and actin. J. Cell Biol..

[36] Ibraghimov-Beskrovnaya O (1992). Primary structure of dystrophin-associated glycoproteins linking dystrophin to the extracellular matrix. Nature.

[37] Holt KH, Campbell KP (1998). Assembly of the sarcoglycan complex. Insights for muscular dystrophy. J. Biol. Chem..

[38] Allen DG, Whitehead NP, Froehner SC (2016). Absence of dystrophin disrupts skeletal muscle signaling: Roles of Ca^2+^, reactive oxygen species, and nitric oxide in the development of muscular dystrophy. Physiol. Rev..

[39] Blake DJ, Weir A, Newey SE, Davies KE (2002). Function and genetics of dystrophin and dystrophin-related proteins in muscle. Physiol. Rev..

[40] Holmberg J, Durbeej M (2013). Laminin-211 in skeletal muscle function. Cell Adhes. Migration.

[41] Fontes-Oliveira CC, Steinz M, Schneiderat P, Mulder H, Durbeej M (2017). Bioenergetic impairment in congenital muscular dystrophy type 1A and Leigh syndrome muscle cells. Sci. Rep..

[42] Capote J (2016). Osteopontin ablation ameliorates muscular dystrophy by shifting macrophages to a pro-regenerative phenotype. J. Cell Biol..

[43] Vanhoutte D (2016). Thrombospondin expression in myofibers stabilizes muscle membranes. eLife.

[44] Quattrocelli M (2017). Genetic modifiers of muscular dystrophy act on sarcolemmal resealing and recovery from injury. PLoS Genet..

[45] Quattrocelli, M., Spencer, M. J. & McNally, E. M. Outside in: The matrix as a modifier of muscular dystrophy. *Biochim. Biophys. Acta* **1864**, 572–579 10.1016/j.bbamcr.2016.12.020 (2016) 10.1016/j.bbamcr.2016.12.020 PMC5262521 28011285

[46] Stenina-Adognravi O (2013). Thrombospondins: Old players, new games. Curr. Opin. Lipidol..

[47] Stenina-Adognravi O (2014). Invoking the power of thrombospondins: Regulation of thrombospondins expression. Matrix Biol. J. Int. Soc. Matrix Biol..

[48] Frolova EG (2014). Control of organization and function of muscle and tendon by thrombospondin-4. Matrix Biol. J. Int. Soc. Matrix Biol..

[49] Brody MJ (2018). Defective flux of thrombospondin-4 through the secretory pathway impairs cardiomyocyte membrane stability and causes cardiomyopathy. Mol. Cell Biol..

[50] Morgan JE (2018). Necroptosis mediates myofibre death in dystrophin-deficient mice. Nat. Commun..

[51] Bencze M, Periou B, Baba-Amer Y, Authier FJ (2019). Immunolabelling myofiber degeneration in muscle biopsies. J. Vis. Exp..

[52] Shimizu Y (2017). Immunoglobulin G (IgG)-based imaging probe accumulates in M1 macrophage-infiltrated atherosclerotic plaques independent of IgG target molecule expression. Mol. Imaging Biol..

[53] Hager M (2008). Cib2 binds integrin alpha7Bbeta1D and is reduced in laminin alpha2 chain-deficient muscular dystrophy. J. Biol. Chem..

[54] de Oliveira BM (2014). Quantitative proteomic analysis reveals metabolic alterations, calcium dysregulation, and increased expression of extracellular matrix proteins in laminin α2 chain-deficient muscle. Mol. Cell Proteom..

[55] Arber S, Caroni P (1995). Thrombospondin-4, an extracellular matrix protein expressed in the developing and adult nervous system promotes neurite outgrowth. J. Cell Biol..

[56] Stenina-Adognravi O, Plow EF (2019). Thrombospondin-4 in tissue remodeling. Matrix Biol. Int. Soc. Matrix Biol..

[57] Frolova EG (2012). Thrombospondin-4 regulates fibrosis and remodeling of the myocardium in response to pressure overload. FASEB J..

[58] Palao T (2018). Thrombospondin-4 mediates cardiovascular remodelling in angiotensin II-induced hypertension. Cardiovasc. Pathol..

[59] Cingolani OH (2011). Thrombospondin-4 is required for stretch-mediated contractility augmentation in cardiac muscle. Circ. Res..

[60] Klaas M (2021). Thrombospondin-4 is a soluble dermal inflammatory signal that selectively promotes fibroblast migration and keratinocyte proliferation for skin regeneration and wound healing. Front. Cell Dev. Biol..

[61] Qian W, Li N, Cao Q, Fan J (2018). Thrombospondin-4 critically controls transforming growth factor beta1 induced hypertrophic scar formation. J. Cell Physiol..

[62] Vainzof M (1996). The sarcoglycan complex in the six autosomal recessive limb-girdle muscular dystrophies. Hum. Mol. Genet..

[63] Chen J (2007). The 16 kDa subunit of vacuolar H+-ATPase is a novel sarcoglycan-interacting protein. Biochim. Biophys. Acta.

[64] Betto R (1999). Ecto-ATPase activity of alpha-sarcoglycan (adhalin). J. Biol. Chem..

[65] Sandona D, Gastaldello S, Martinello T, Betto R (2004). Characterization of the ATP-hydrolysing activity of alpha-sarcoglycan. Biochem. J..

[66] Stenina OI, Topol EJ, Plow EF (2007). Thrombospondins, their polymorphisms, and cardiovascular disease. Arterioscler. Thromb. Vasc. Biol..

[67] Kirk JA, Cingolani OH (2016). Thrombospondins in the transition from myocardial infarction to heart failure. J. Mol. Cell Cardiol..

[68] Schips TG (2019). Thrombospondin-3 augments injury-induced cardiomyopathy by intracellular integrin inhibition and sarcolemmal instability. Nat. Commun..

[69] Frolova EG (2010). Thrombospondin-4 regulates vascular inflammation and atherogenesis. Circ. Res..

[70] Gawlik K, Miyagoe-Suzuki Y, Ekblom P, Takeda S, Durbeej M (2004). Laminin alpha1 chain reduces muscular dystrophy in laminin alpha2 chain deficient mice. Hum. Mol. Genet..

[71] Gawlik KI (2006). Laminin alpha1 chain mediated reduction of laminin alpha2 chain deficient muscular dystrophy involves integrin alpha7beta1 and dystroglycan. FEBS Lett..

