# Supplementary material for: Thrombospondin-4 deletion does not exacerbate muscular dystrophy in β-sarcoglycan-deficient and laminin α2 chain-deficient mice
Source: Sci Rep. 2024 Jun 26;14:14757. doi: 10.1038/s41598-024-65473-8 (PMC11208443; doi:10.1038/s41598-024-65473-8)
Supplement: Supplementary file 1 — Supplementary Figures. [file 41598_2024_65473_MOESM1_ESM.docx]

**Thrombospondin-4 does not act as a genetic modifier of limb-girdle muscular dystrophy type 2E and LAMA2-related muscular dystrophy**

Paula Zarén and Kinga I. Gawlik*

Muscle Biology Unit, Department of Experimental Medical Science, Lund University, Lund, Sweden

*Corresponding author:

Kinga I. Gawlik

Muscle Biology Unit

Department of Experimental Medical Science

BMC C12, Lund University

221 84 Lund, Sweden

Tel : 0046 46 2220813

Email: [kinga.gawlik@med.lu.se](mailto:kinga.gawlik@med.lu.se)


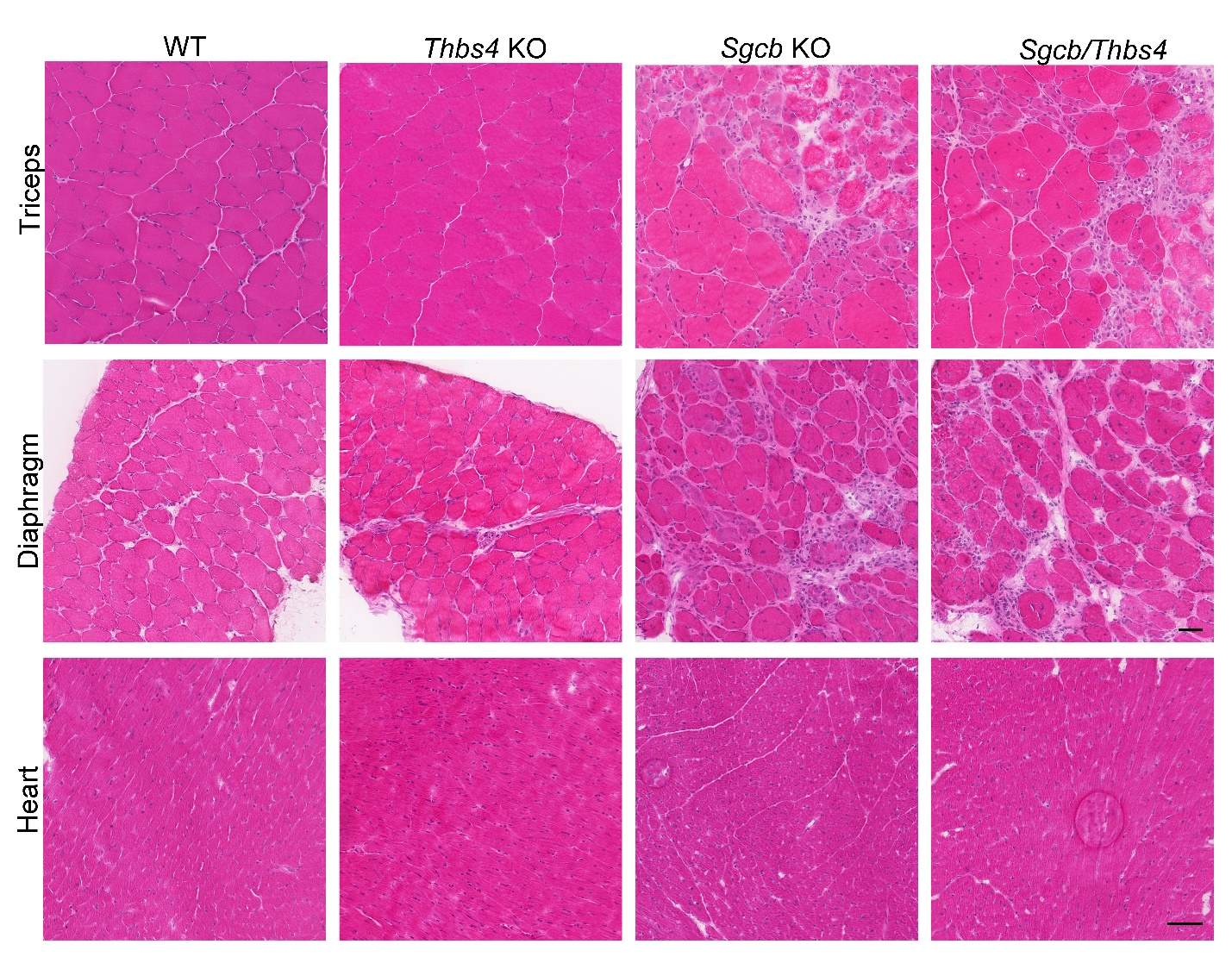


Supplementary Fig. S1. Hematoxylin and eosin staining of triceps, diaphragm and heart muscles isolated from 3-month-old WT, *Thbs4* KO, *Sgcb* KO, and *Sgcb/Thbs4* mice. *Thbs4* KO muscles do not exhibit histopathological signs of muscular dystrophy. Dystrophic features are equally pronounced in triceps and diaphragm from *Sgcb* KO and *Sgcb/Thbs4* animals. Heart muscle isolated from 3-month-old WT, *Thbs4* KO, *Sgcb* KO, and *Sgcb/Thbs4* mice does not show histopathological changes in any of the studied groups at this age. Bars, 50 μm.


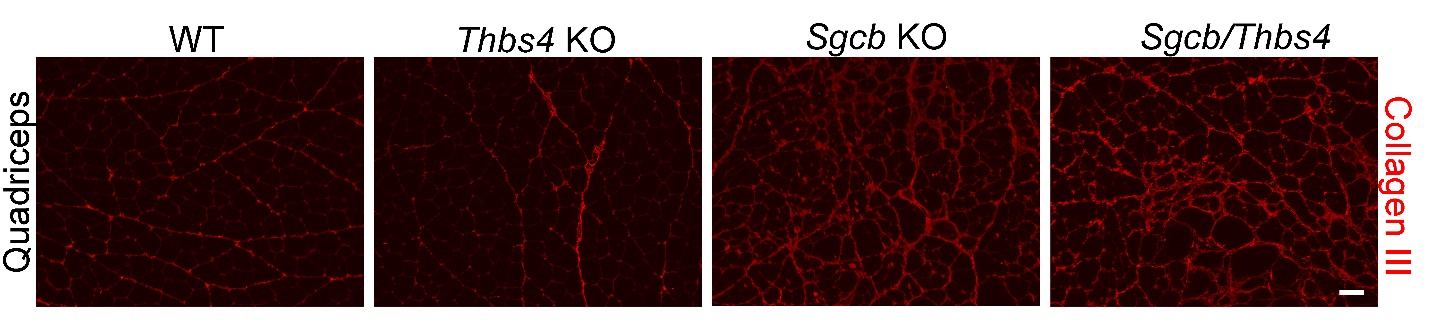


Supplementary Fig. S2 Elevated production of collagen III in 3-month-old *Sgcb* KO and *Sgcb/Thbs4* muscle. Collagen III immunostaining demonstrates the same pattern of increased fibrosis in both dystrophic strains.


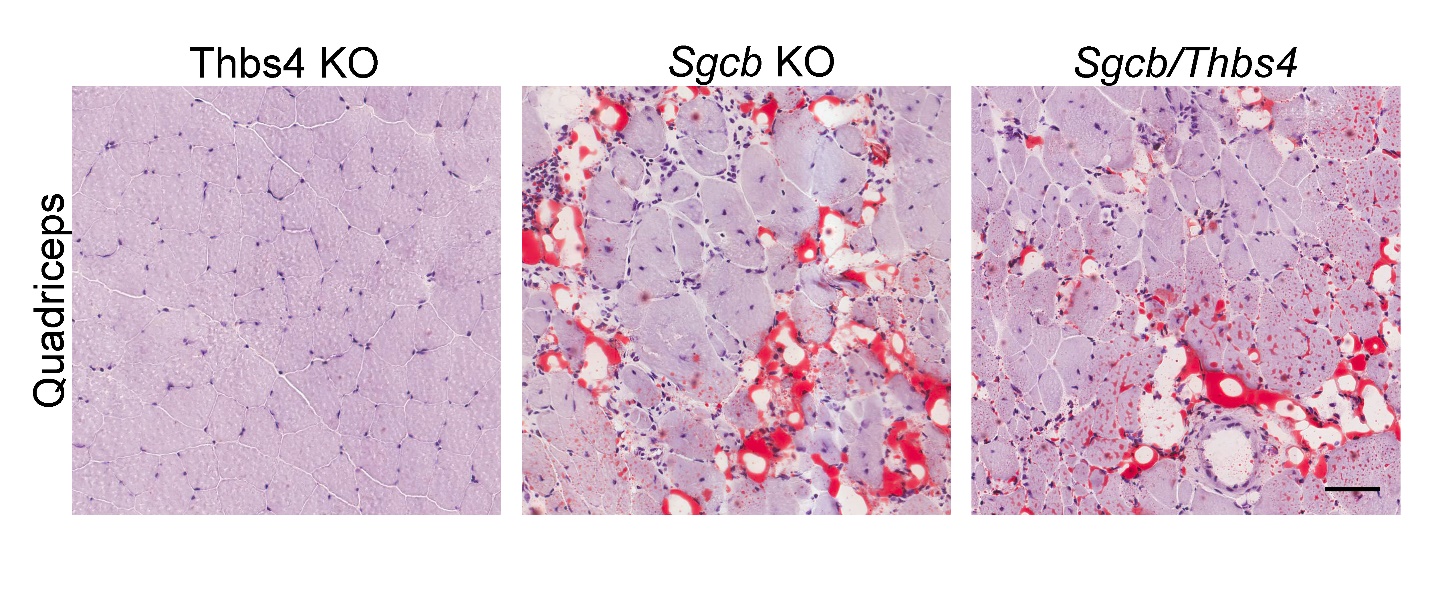


Supplementary Fig. S3 Oil Red O staining of quadriceps muscle from 15-month-old *Thbs4* KO, *Sgcb* KO, and *Sgcb/Thbs4* mice to visualize fat cells. *Sgcb* KO and *Sgcb/Thbs4* muscle display similar amount of fatty infiltrates. Bar, 50 μm.


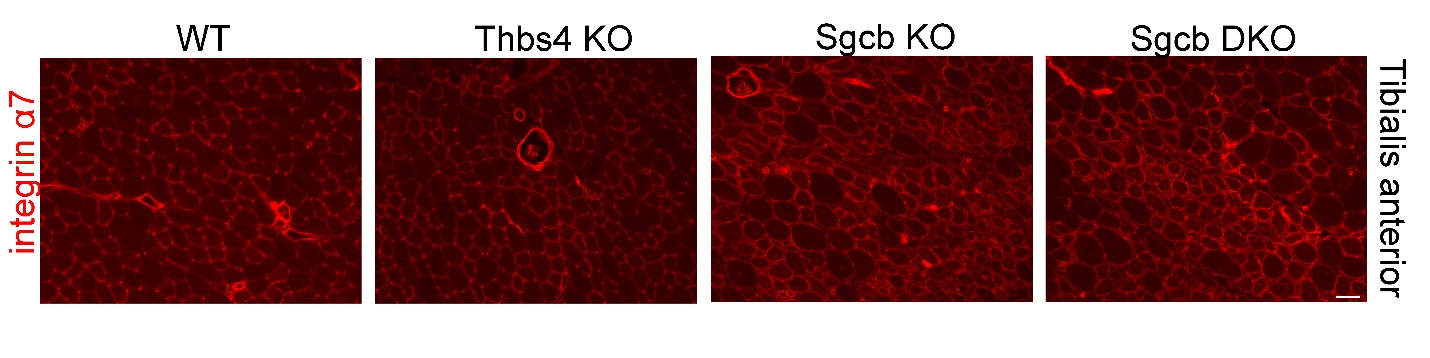


Supplementary Fig. S4 Upregulation of integrin α7 in tibialis anterior of *Sgcb* KO and *Sgcb/Thbs4* mice. Bar, 50 μm.


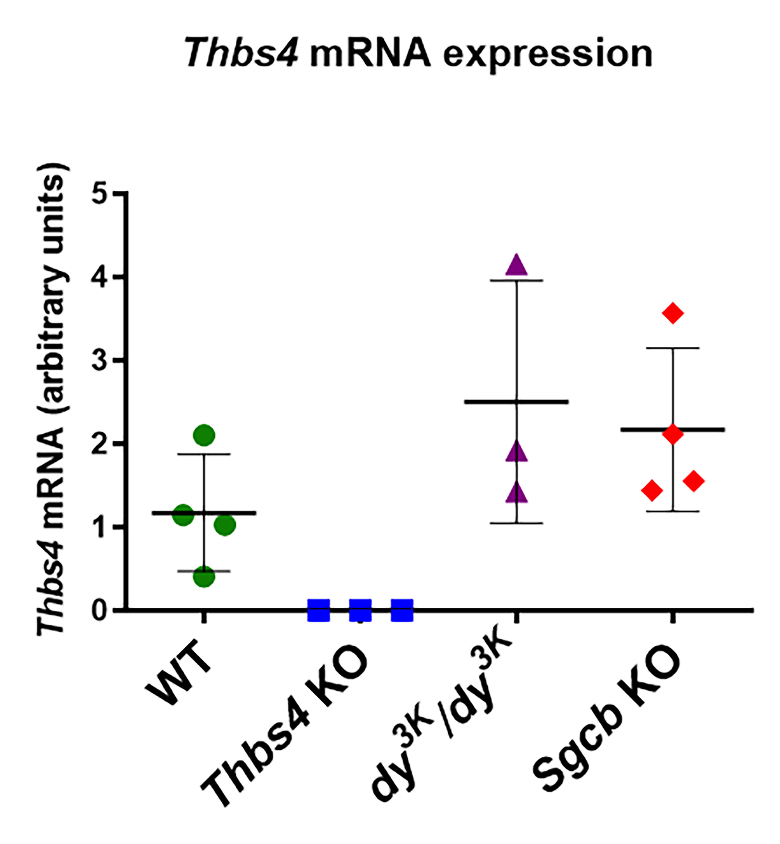


Supplementary Fig. S5 qPCR analysis of mRNA from wild-type, *Thbs4* KO, *dy^3K^/dy^3K^* and *Sgcb* KO quadriceps revealed no significant increase of Thbs4 transcript in dystrophic muscles compared to wild-type muscle (p=0.2203; p=0.3429, respectively; one-way ANOVA followed by Tukey’s multiple comparison test). As expected, no expression of Thbs4 mRNA was detected in *Thbs4* KO.
